# Single-cell epigenomics reveals mechanisms of human cortical development

**DOI:** 10.1038/s41586-021-03209-8

**Published:** 2021-10-06

**Authors:** Ryan S. Ziffra, Chang N. Kim, Jayden M. Ross, Amy Wilfert, Tychele N. Turner, Maximilian Haeussler, Alex M. Casella, Pawel F. Przytycki, Kathleen C. Keough, David Shin, Derek Bogdanoff, Anat Kreimer, Katherine S. Pollard, Seth A. Ament, Evan E. Eichler, Nadav Ahituv, Tomasz J. Nowakowski

**Affiliations:** 1 Department of Anatomy, University of California, San Francisco, San Francisco, CA USA; 2 Department of Psychiatry, University of California, San Francisco, San Francisco, CA USA; 3 Eli and Edythe Broad Center for Regeneration Medicine and Stem Cell Research, University of California, San Francisco, San Francisco, CA USA; 4 Department of Bioengineering and Therapeutic Sciences, University of California, San Francisco, San Francisco, CA USA; 5 Institute for Human Genetics, University of California, San Francisco, San Francisco, CA USA; 6 Department of Genome Sciences, University of Washington School of Medicine, Seattle, WA USA; 7 Department of Genetics, Washington University School of Medicine, St. Louis, MO USA; 8 Genomics Institute, University of California, Santa Cruz, Santa Cruz, CA USA; 9 Institute for Genome Sciences, University of Maryland School of Medicine, Baltimore, MD USA; 10 Medical Scientist Training Program, University of Maryland School of Medicine, Baltimore, MD USA; 11 Gladstone Institutes, San Francisco, CA USA; 12 Institute for Computational Health Sciences, University of California, San Francisco, San Francisco, CA USA; 13 University of California, San Francisco, San Francisco, CA USA; 14 Department of Electrical Engineering and Computer Sciences, University of California, Berkeley, Berkeley, CA USA; 15 Center for Computational Biology, University of California, Berkeley, Berkeley, CA USA; 16 Department of Epidemiology and Biostatistics, University of California, San Francisco, San Francisco, CA USA; 17 Quantitative Biology Institute, University of California, San Francisco, San Francisco, CA USA; 18 Chan Zuckerberg Biohub, San Francisco, San Francisco, CA USA; 19 Department of Psychiatry, University of Maryland School of Medicine, Baltimore, MD USA; 20 Howard Hughes Medical Institute, University of Washington, Seattle, WA USA

**Keywords:** Developmental neurogenesis, Neural patterning

## Abstract

During mammalian development, differences in chromatin state coincide with cellular differentiation and reflect changes in the gene regulatory landscape^1^. In the developing brain, cell fate specification and topographic identity are important for defining cell identity^2^ and confer selective vulnerabilities to neurodevelopmental disorders^3^. Here, to identify cell-type-specific chromatin accessibility patterns in the developing human brain, we used a single-cell assay for transposase accessibility by sequencing (scATAC-seq) in primary tissue samples from the human forebrain. We applied unbiased analyses to identify genomic loci that undergo extensive cell-type- and brain-region-specific changes in accessibility during neurogenesis, and an integrative analysis to predict cell-type-specific candidate regulatory elements. We found that cerebral organoids recapitulate most putative cell-type-specific enhancer accessibility patterns but lack many cell-type-specific open chromatin regions that are found in vivo. Systematic comparison of chromatin accessibility across brain regions revealed unexpected diversity among neural progenitor cells in the cerebral cortex and implicated retinoic acid signalling in the specification of neuronal lineage identity in the prefrontal cortex. Together, our results reveal the important contribution of chromatin state to the emerging patterns of cell type diversity and cell fate specification and provide a blueprint for evaluating the fidelity and robustness of cerebral organoids as a model for cortical development.

## Abstract (web summary)

Analysis of chromatin state at a single-cell level in samples of developing human forebrain demonstrate both cell-type-specific and region-specific changes during neurogenesis.

## Main

**Fig. 1:**
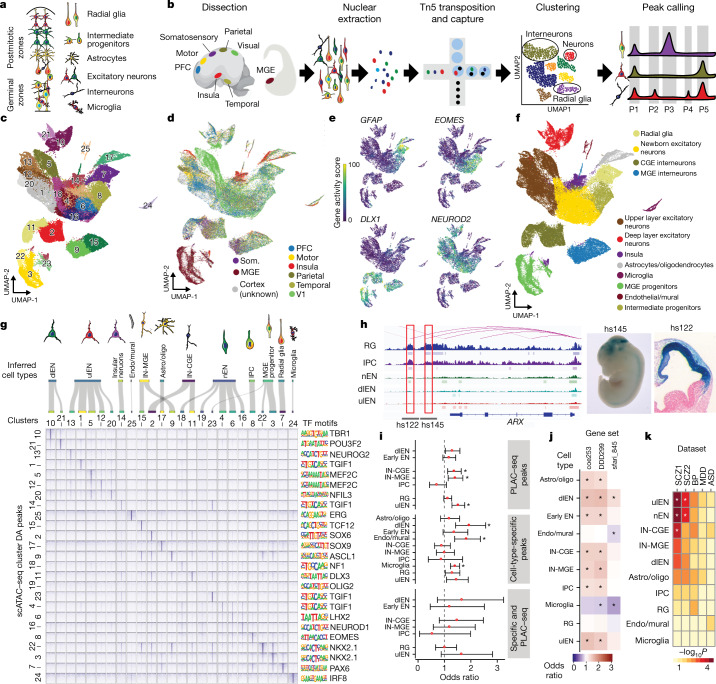
Single-cell chromatin state atlas of the developing human brain. **a**, Schematic cross-section of developing cortex, highlighting major cell types. **b**, Experimental workflow. **c**, Uniform manifold approximation and projection (UMAP) of primary scATAC-seq cells (*n* = 6 individuals, 77,354 cells) coloured by clusters. **d**, UMAP projection of primary scATAC-seq cells coloured by brain region. Som., somatosensory cortex. **e**, UMAP projections of gene activity scores for *GFAP* (marking glia), *EOMES* (IPCs), *DLX1* (interneuron lineage cells), and *NEUROD2* (excitatory lineage cells). **f**, UMAP projection of primary scATAC-seq cells coloured by broad cell type. **g**, Top, Sankey plot linking scATAC-seq clusters and cell-type predictions. Endo, endothelial; astro/oligo, astrocyte/oligodendrocyte precursors; nEN, newborn excitatory neurons. Bottom left, pile-ups of ATAC-seq signal for each cluster within sets of the top 1,000 enriched peaks for each cluster (Fisher’s exact, two-sided). Pile-ups are centred on peak centres and the ±10-kb flanking region is depicted. Bottom right, significantly enriched transcription factor (TF) motifs for each cluster-specific peak set (hypergeometric test, one-sided). **h**, Left, predicted enhancer–gene interactions for RGs (pink curves) overlayed with ATAC-seq signal tracks and peaks for RGs, IPCs, nENs, dlENs, and ulENs. Red boxes highlight predicted enhancers of *ARX* that overlap with validated VISTA forebrain enhancers^25^. Right, LacZ staining marking regions of enhancer activity for the enhancer candidates hs122 and hs145 in embryonic day (E)12.5 mouse embryos, depicting activity in the forebrain^6,55^ (images reproduced with permission from VISTA Enhancer Browser; https://enhancer.lbl.gov/). At E12.5, embryos have an average crown-rump length of 8.6 mm. **i**, Enrichment and depletion of peaks that overlap with promoter-interacting regions^21^, cell-type-specific peaks, and peaks that meet both criteria in copy number variant (CNV) regions enriched in children with NDD^30^ (*n* = 70 NDD-associated CNVs; Fisher’s exact, two-sided, *P* < 0.05). Asterisks, Bonferroni-corrected significance. Error bars, 95% confidence interval (CI). **j**, Enrichment and depletion of peaks that overlap with predicted enhancers in promoter and gene regions of genes associated with ASD and NDD, including genes enriched in de novo non-coding mutations (SFARI845; https://gene.sfari.org/database/human-gene/, DDD299^56^, COE253^30^) (Fisher’s exact, two-sided). Asterisks, Bonferroni-corrected significance. **k**, Heat map of heritability enrichment based on linkage disequilibrium (LD) score regression analysis of genome-wide association study (GWAS) summary statistics in cell-type-specific peak sets coloured by −log_10_(*P*). Asterisks, FDR < 0.05. From left to right: Psychiatric Genomics Consortium (PGC) schizophrenia (SCZ) GWAS^27^; an additional PGC schizophrenia GWAS^32^; PGC bipolar (BP) disorder^35^; PGC major depressive disorder (MDD) GWAS^34^; PGC ASD GWAS^33^.

Cell types of the cerebral cortex (Fig. 1a) have traditionally been classified on the basis of a handful of morphological, anatomical, and physiological features. Recent innovations in single-cell transcriptomics, including single-cell RNA sequencing (scRNA-seq), have enabled massively parallel profiling of thousands of molecular features in individual cells and uncovered distinctions among closely related cell types, such as excitatory neurons located in different areas of the cerebral cortex^2,4^. Despite these advances, the developmental mechanisms that underlie the emergence of distinct neuronal lineages in the human cerebral cortex remain largely unknown^5^.

Chromatin state defines the functional architecture of the genome by modulating the accessibility of gene regulatory elements, such as enhancers, which serve as binding sites for transcriptional regulators. During development, sequential cascades of transcription factors progressively remodel and refine differential patterns of chromatin accessibility across distinct cell types^6,7^. Identifying the highly dynamic and cell-type-specific patterns of enhancer activity could provide critical insights into the molecular mechanisms that govern cell fate specification. Although chromatin accessibility represents a fundamental feature of cell identity, relatively few studies have profiled chromatin state changes during brain development^1,8–11^. Recently, innovations in single-cell genomics have enabled scalable profiling of chromatin state with cellular resolution using scATAC-seq^12^. In the developing mouse brain^13^, scATAC-seq has revealed highly dynamic changes in chromatin accessibility that underlie neurodevelopmental processes. It will be necessary to extend these studies to human primary tissue in order to better understand how mutations in non-coding regulatory elements, including human-specific neurodevelopmental enhancers, interfere with normal developmental processes and contribute to genetic burden in psychiatric neurodevelopmental disorders^14,15^.

## Chromatin states of the developing brain

To characterize the chromatin state landscape of the developing human brain at single-cell resolution, we performed scATAC-seq on primary samples of human forebrain at mid-gestation (*n* = 6 individuals), including samples of dorsolateral prefrontal cortex (PFC), primary visual cortex (V1), primary motor cortex (M1), primary somatosensory cortex, dorsolateral parietal cortex, temporal cortex, insular cortex, and the medial ganglionic eminence (MGE) (Fig. 1b, Supplementary Table 1).

**Extended Data Fig. 1:**
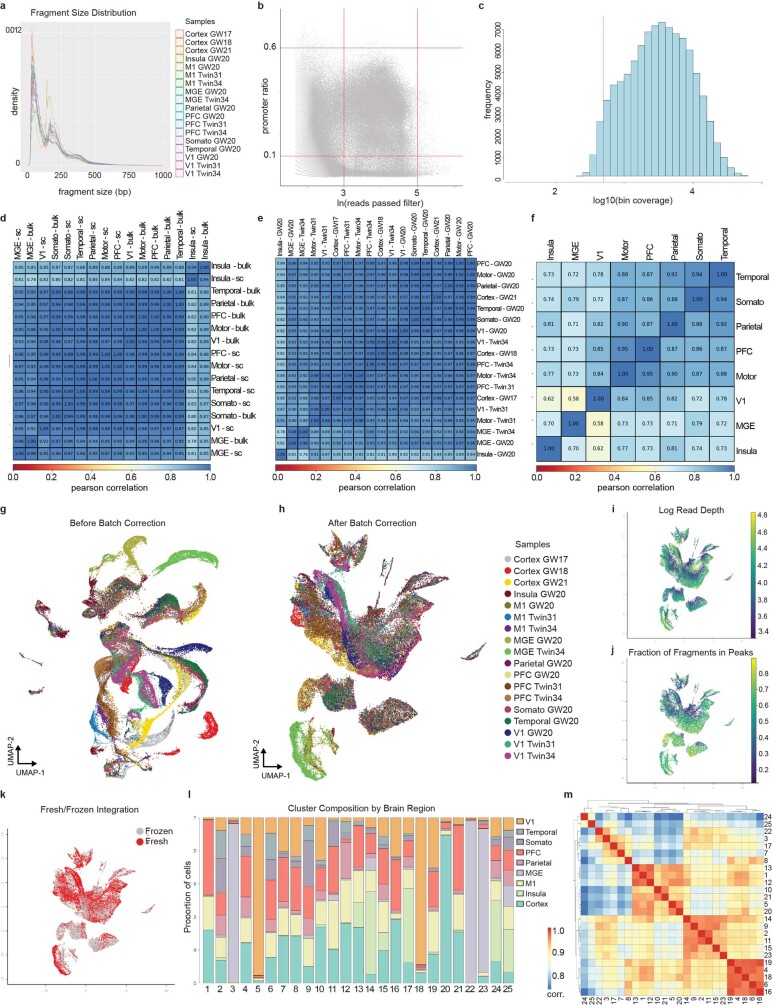
Batch correction and quality control metrics for primary scATAC-seq data. **a**, Density curve of fragment size distribution for deduplicated, uniquely mapped fragments that passed quality filters for each sample. **b**, Plot of promoter ratio against ln(read depth) for all cell barcodes detected. Cells were included in downstream analysis that had a promoter ration of 10–60% and ln(read depth) of 3–5. Red lines indicate upper and lower thresholds. **c**, Histogram of cellular coverage in 5-kb genomic bins for all cells. Cells with reads in fewer than 500 bins were removed from downstream analysis. **d**, Heat map of all-by-all Pearson correlations between all samples that had bulk libraries prepared in parallel. Bulk and aggregate single-cell libraries for each sample are included. Correlation was calculated in the space of the merged primary peak set (*n* = 459,953 peaks). **e**, Heat map of all-by-all Pearson correlations between all aggregate single-cell libraries. Correlation was calculated in the space of the merged primary peak set (*n* = 459,953 peaks). **f**, Heat map of all-by-all correlations between primary single-cell data aggregated by area of origin. Correlation was calculated in the space of the merged primary peak set (*n* = 459,953 peaks). **g**, **h**, UMAP projections of all primary scATAC-seq cells before (**g**) and after (**h**) batch correction coloured by sample (see Methods). **i**, UMAP projection of log(read depth) for deduplicated, uniquely mapped reads that passed quality filters on all primary scATAC-seq cells. **j**, UMAP projection of fraction of fragments in peaks on all primary scATAC-seq cells. **k**, UMAP projection of all primary scATAC-seq cells coloured by condition (fresh or frozen). **l**, Bar plot depicting the proportion of cells from each brain region for each Leiden cluster for all primary scATAC-seq cells. **m**, Heat map of all-by-all Pearson correlations between clusters. Correlation is calculated in the space of all primary peaks (*n* = 459,953).

**Extended Data Fig. 2:**
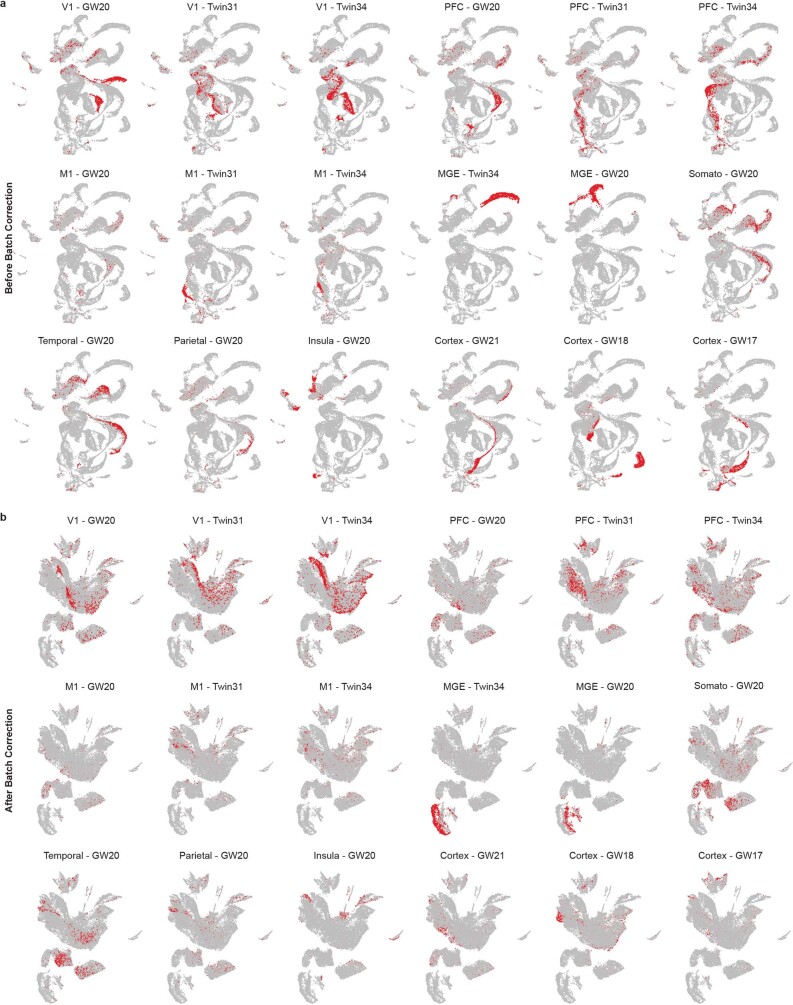
Batch Correction of primary scATAC-seq samples. **a**, UMAP projections of all primary scATAC-seq cells that passed quality control before batch correction with all cells from each sample coloured in red. **b**, UMAP projections of all primary scATAC-seq cells that passed quality control after batch correction with all cells from each sample coloured in red.

We generated data from 77,354 cells that passed quality control criteria (Methods, Extended Data Fig. 1a–c). Aggregated signal from single-cell libraries correlated strongly with bulk ATAC-seq libraries generated in parallel (Extended Data Fig. 1d), and data from biological replicates were highly correlated (Extended Data Fig. 1e, f). To reduce the dimensionality of the dataset, we performed latent semantic indexing followed by singular value decomposition (see Methods). Batch correction was performed using the deep neural network-based tool scAlign^16^ to correct for technical sources of variance, including individual variation and processing method (Extended Data Figs. 1g–k, 2, Methods). We identified 25 distinct clusters using the Leiden community detection algorithm (Fig. 1c, Extended Data Fig. 1l, m; Methods). This analysis robustly separated cortical and subcortical (MGE) cells (Fig. 1d).

**Extended Data Fig. 3:**
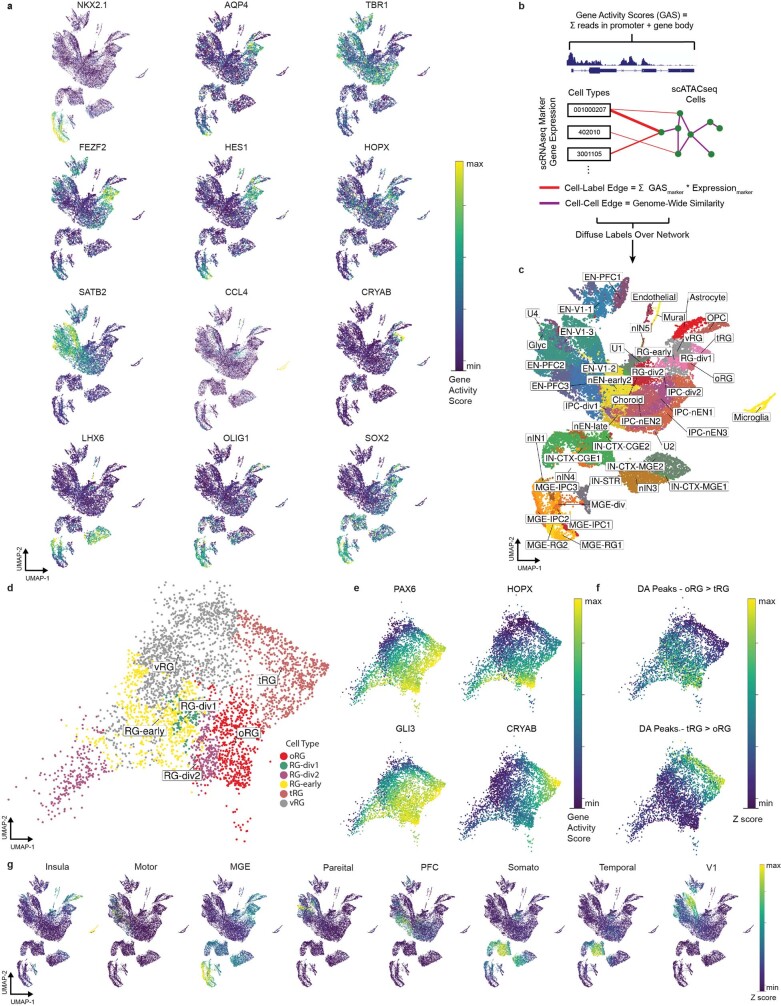
Gene activity scores correlate with cell type-specific expression of marker genes. **a**, UMAP projections of all primary scATAC-seq cells coloured by gene activity score. From top left to bottom right: *NKX2.1* (also known as *NKX2-1*) marking MGE cells, *AQP4* marking glia/astrocytes, *TBR1* marking excitatory neurons, *FEZF2* marking deep layer excitatory neurons, *HES1* marking radial glia, *HOPX* marking outer radial glia, *SATB2* marking upper layer excitatory neurons, *CCL4* marking microglia, *CRYAB* marking truncated radial glia, *LHX6* marking MGE-derived interneurons, *OLIG1* marking oligodendrocyte precursors, and *SOX2* marking radial glia. **b**, Schematic of the CellWalker algorithm^17^ used to assign cell-type labels to scATAC-seq cells based on integration with scRNA-seq data. **c**, UMAP projection of cell-type labels assigned by CellWalker. **d**, UMAP projection of radial glia with cell-type assignments from CellWalker. **e**, UMAP projections of gene activity scores for *PAX6* and *GLI3*, genes that are ubiquitously expressed in radial glia; *HOPX*, an oRG-specific gene^2^; and *CRYAB*, a tRG-specific gene^2^. **f**, UMAP projection of *Z-*scores of enrichment of oRG- and tRG-specific peaks (Fisher’s exact, two-sided test, *P* < 0.05). **g**, UMAP projections of *Z*-scores of enrichment of area-specific peaks for each area in all primary scATAC-seq cells (Fisher’s exact, two-sided test, *P* < 0.05).

To infer the identities of cell clusters, we calculated ‘gene activity scores’, which represent a proxy for gene expression^13^, by summing fragments in the gene body and promoter regions (Methods). We identified the major cell classes, including radial glia (RGs), intermediate progenitor cells (IPCs), deep layer (cortical layers V–VI) excitatory neurons (dlENs), upper layer (cortical layers II–IV) excitatory neurons (ulENs), MGE- and CGE-derived cortical interneurons (IN-MGEs and IN-CGEs, respectively), insular neurons, progenitors from the MGE, microglia, oligodendrocyte progenitor cells (OPCs), endothelial cells, and mural cells (Fig. 1e, f, Extended Data Fig. 3a). In addition, we used CellWalker^17^ to assign cell-type labels to scATAC-seq cells on the basis of previously published scRNA-seq data (Methods, Extended Data Fig. 3b, c). CellWalker identified cell types at a finer resolution, including subtypes of broader cell classes. For example, RGs form a single cluster, but CellWalker identified multiple RG subtypes (dividing (dRGs), ventricular (vRGs), outer (oRGs), and truncated (tRGs)) as sub-clusters (Extended Data Fig. 3d). Furthermore, we could identify differentially accessible peaks between two subtypes (tRGs and oRGs) that differ in their expression of *CRYAB* and *HOPX*, respectively^2^, suggesting that scATAC-seq is sensitive enough to distinguish cellular subtypes at high resolution (Extended Data Fig. 3e, f, Supplementary Tables 12, 13, 22).

## Identifying cell-type-specific enhancers

**Extended Data Fig. 4:**
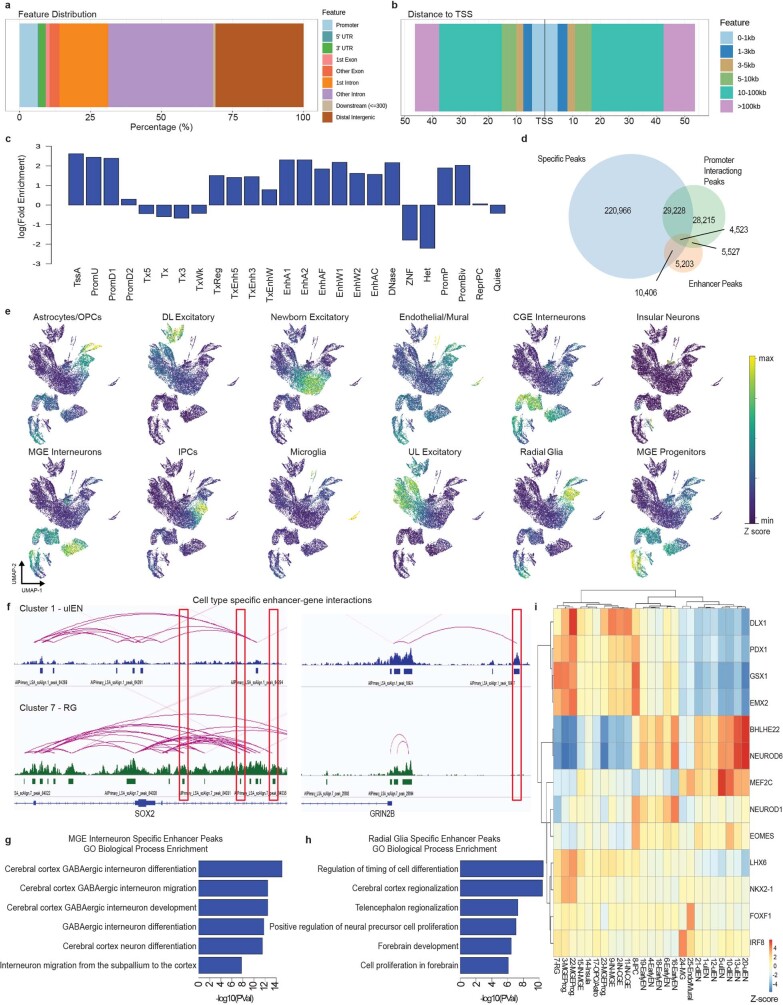
Annotation of primary scATAC-seq peaks. **a**, Distribution of primary scATAC-seq peaks in genomic features. **b**, Distribution of primary scATAC-seq peaks around transcription start sites. **c**, Bar plot of log(fold enrichment) of primary scATAC-seq peaks in chromatin states. Chromatin states defined by the 25-state model from Roadmap Epigenomics^18^ (see Methods). **d**, Intersection of cell-type-specific peaks (Fisher’s exact, two sided, FDR < 0.05), predicted enhancer peaks (see Methods), and peaks overlapping promoter-interacting regions identified by H3K4me3 PLAC–seq^21^. **e**, UMAP projections of *Z*-scores of enrichment of cell-type-specific peaks (Fisher’s exact, two-sided, FDR < 0.05) for each broad cell type. **f**, Browser tracks highlighting cell-type-specific predicted enhancers. Left, highlighting predicted enhancers linked to *SOX2* in RGs that are not present in ulENs. Right, highlighting a predicted enhancer for *GRIN2B* that is present in ulENs and not RGs. **g**, **h**, Bar plots of −log_10_(*P*) of gene ontology biological processes that are enriched in cell-type-specific predicted enhancers of MGE-derived interneurons (**g**) and RGs (**h**) (see Methods). **i**, Heat map of *Z*-scores of transcription factor motif enrichments of key lineage-associated transcription factors in each cluster.

To identify candidate gene regulatory elements, we called peaks on aggregate single cells from each broad cell class (Methods). We subsequently merged overlapping peaks to a total union set of 459,953 peaks (Supplementary Tables 2, 3). Annotation of our peak set in genomic features showed enrichment in intronic and distal intergenic regions and in the flanking regions of transcription start sites, suggesting enrichment on gene regulatory elements, such as enhancers (Extended Data Fig. 4a, b). We intersected our peak set with the imputed 25-state chromatin model from Roadmap Epigenomics^18^ and found strong enrichment for promoter and enhancer states and depletion of transcribed, heterochromatin, and quiescent states (Methods, Extended Data Fig. 4c, Supplementary Table 14). We identified cell-type-specific differentially accessible peaks for each cell type, resulting in a set of 265,123 peaks, with most cell types having on the order of thousands of specific peaks (Fisher’s exact test, false discovery rate (FDR) <0.05; Fig. 1g, Extended Data Fig. 4d, e, Supplementary Tables 4, 6, 7). In addition, we identified peaks that are differentially accessible between the eight brain regions used in this study (Extended Data Fig. 3g, Supplementary Table 8). To identify putative enhancers in our dataset, we integrated our ATAC-seq peaks with cleavage under targets and tagmentation (CUT&Tag) data for acetylation of lysine 27 on histone H3 (H3K27ac) generated from similar samples (Methods), Hi-C chromosome conformation capture data generated from developing human cortex^19^, and gene expression data^2^, and used the activity-by-contact algorithm^20^ to predict enhancer–gene interactions (Methods) for all cortical cell types. In total, we predicted 25,659 gene-linked enhancers across the whole dataset (Extended Data Fig. 4d, f, Supplementary Table 5). We intersected our peaks with promoter-interacting regions identified using trimethylation of lysine 4 on histone H3 (H3K4me3) proximity ligation-assisted chromatin immunoprecipitation with sequencing (PLAC–seq) on sorted cells from developing human cortex^21^, and found 67,493 peaks and 10,050 predicted enhancers with physical evidence of promoter interaction (Extended Data Fig. 4d, Supplementary Table 15). Genes linked to predicted cell-type-specific enhancers were enriched for biological processes strongly associated with cell-type identity (Methods, Extended Data Fig. 4g, h).

**Extended Data Fig. 5:**
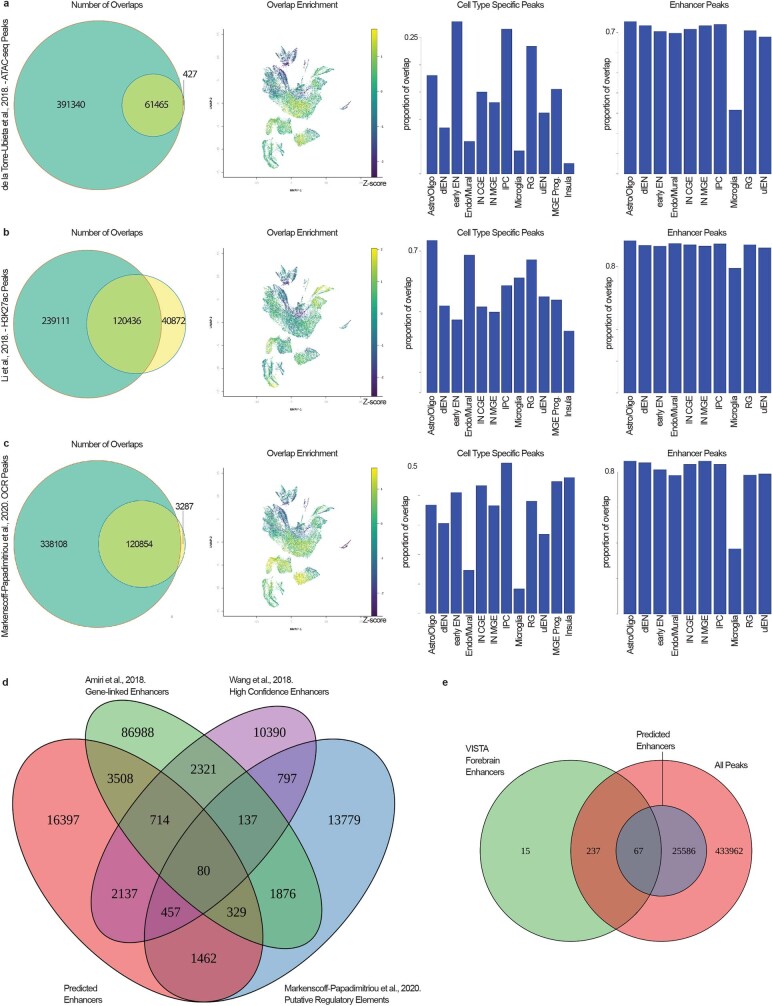
scATAC-seq peaks overlap with previously annotated bulk ATAC-seq peaks and validated forebrain enhancers. **a**–**c**, Overlap of all primary peaks with the peak set from de la Torre-Ubieta et al.^9^ (**a**), Li et al.^22^ (**b**), and Markenscoff-Papadimitriou et al.^23^ (**c**). Left to right: Venn diagram of overlaps, UMAP projection of *Z*-scores of enrichment of overlapping peaks in all primary scATAC-seq cells, bar plot of proportions of cell-type-specific peaks (Fisher’s exact, two-sided, FDR < 0.05) present in overlapping set, bar plot of proportions of predicted enhancers for each cell type (see Methods) present in the overlapping set. **d**, Venn diagram of overlap of all predicted enhancers (see Methods) with gene-linked enhancers from Amiri et al.^11^, high confidence enhancers from Wang et al.^24^, and putative regulatory elements from Markenscoff-Papadimitriou et al.^23^. **e**, Venn diagram of overlaps of VISTA forebrain enhancers^25^ with all primary scATAC-seq peaks, and all predicted enhancers (see Methods).

To further support our annotations, we intersected our peak set with publicly available datasets generated from human cortical tissue samples^9,22,23^ (Extended Data Fig. 5a–c, Supplementary Table 15). We found that scATAC-seq recovered most of the peaks that were annotated using bulk tissue datasets, and also recovered many putative cell-type-specific peaks that were not captured in bulk datasets, especially those enriched in rarer cell populations such as microglia and endothelial cells (Supplementary Table 4). We intersected our predicted enhancers with other enhancer predictions derived from previously published datasets^11,23,24^. Unexpectedly, we did not find strong concordance between predicted enhancers from these studies (Extended Data Fig. 5d). Among functionally validated forebrain enhancers^25^, the majority (304 out of 319) overlapped chromatin accessibility peaks, but only 67 overlapped enhancers predicted using activity-by-contact (Fig. 1h, Extended Data Fig. 5e). Together, these analyses suggest that scATAC-seq is a robust method for detecting chromatin accessibility patterns from heterogeneous tissue samples. However, limited overlap of predicted enhancers with previously published studies indicates that a better understanding of the relevant feature set for computationally predicting regulatory potential is urgently needed.

To characterize the regulatory ‘grammar’ of cell types, we calculated the enrichment of known transcription factor binding motifs in cell-type-specific peak sets (Methods, Fig. 1g, Supplementary Table 20). Transcription factor motif enrichments were strongly associated with cell-type annotations from marker gene body enrichments. To examine transcription factor motif enrichments at the single-cell level, we used ChromVAR^26^ (Methods) and found substantial agreement with top motif enrichments for each cluster (Extended Data Fig. 4i). Together, these findings ascertain that scATAC-seq identifies chromatin accessibility patterns consistent with known transcription factor expression patterns across cell types and provides a roadmap towards the discovery of a transcription factor ‘code’ that underlies cell lineage and cell fate specification.

## Disease risk in the regulatory landscape

**Extended Data Fig. 6:**
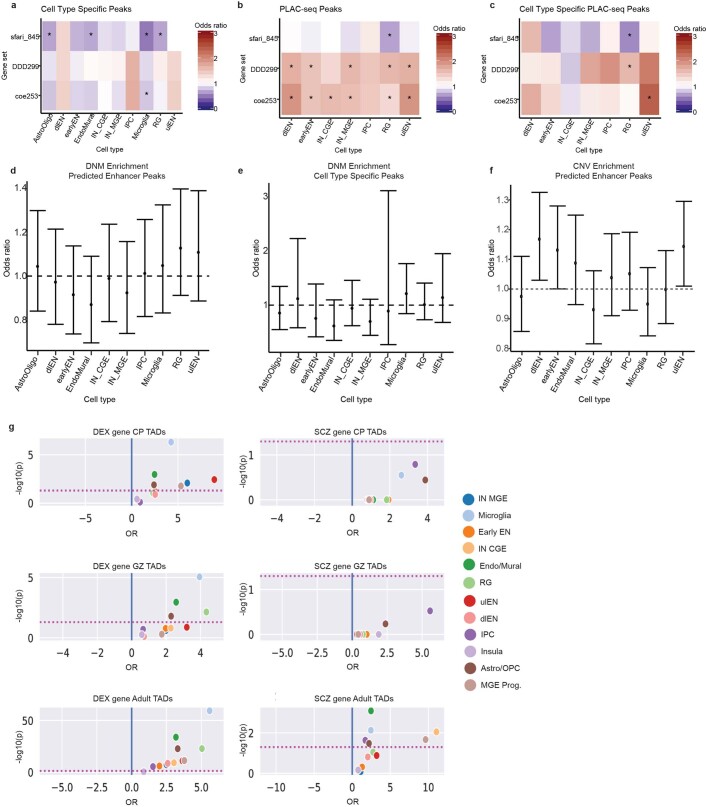
Enrichment and depletion of disease associated variants in scATACseq peaks. **a**–**c**, Enrichment and depletion of cell-type-specific peaks (**a**), peaks that overlap with H3K4me3 PLAC–seq interactions^21^ (**b**) and cell-type-specific peaks that overlap with H3K4me3 PLAC–seq interactions^21^ (**c**) (Fisher’s exact, two-sided, FDR < 0.05) in promoter and gene regions of genes associated with ASD and NDD, including genes enriched in DNMs (SFARI845, DDD29960^56^, COE25361^30^). Asterisks indicate tests that pass Bonferroni significance. **d**, Enrichment and depletion of DNMs in predicted enhancer peaks for each cell type in ASD probands compared with unaffected siblings. DNM data from a total of 2767 probands and 1855 unaffected siblings were included in the analysis. No tests reached Bonferroni significance. Bars represent 95% CI. **e**, Enrichment and depletion of DNMs in cell-type-specific peaks for each cell type in ASD probands compared with unaffected siblings. DNM data from a total of 2,767 probands and 1,855 unaffected siblings were included in the analysis. No tests reached Bonferroni significance. Bars represent 95% CI. **f**, Enrichment and depletion of predicted enhancer peaks in copy number variant (CNV) regions enriched in paediatric cases of NDD^30^ (*n* = 70 NDD-associated CNVs). No tests reached Bonferroni significance. Bars represent 95% CI. **g**, Enrichment of cell-type-specific enhancers located in TADs with neurodevelopmental disease-associated genes^31^ (left) and schizophrenia-associated genes^24^ (right). Three distinct sets of TADs were used (top to bottom): TADs defined in the cortical plate (CP) of developing human cortex, TADs defined in the germinal zone (GZ) of developing human cortex, and TADs defined in adult cortex^19^.

Mutations in non-coding genomic regions, as well as de novo loss-of-function mutations in chromatin regulators, have been implicated in a wide range of neurodevelopmental and psychiatric disorders, including schizophrenia^27^ and autism spectrum disorder (ASD)^3,28,29^. Cellular-resolution datasets of chromatin state across developmental stages and differentiation states may provide an important link between these mutations and selective vulnerabilities among the diverse cell types of the developing human brain, as was seen with recent studies using single-cell transcriptomic data^3,22^. Towards that end, we intersected cell-type-specific ATAC-seq peaks and putative enhancers with disease-linked common and rare non-coding variants (Methods). We first intersected cell-type-specific peak sets, predicted enhancers, and peaks that overlapped promoter-interacting regions^21^ with genomic regions that were enriched for copy number variants in individuals with developmental delay^30^; we identified significant enrichment in dlEN, endothelial/mural, and microglia-specific peaks, as well as peaks that overlapped promoter-interacting regions in interneurons (Fig. 1i, Extended Data Fig. 6f; Fisher’s exact, two-sided, *P* < 0.05). Because such regions do not provide specificity with respect to individual regulatory elements or genes, we next tested for enrichment of cell-type-specific peaks, predicted enhancers, and peaks that overlapped promoter-interacting regions in the flanking regions of genes associated with ASD and neurodevelopmental delay (NDD) and identified peak sets that were significantly enriched or depleted in these regions for most cell types (Fig. 1j, Extended Data Fig. 6a–c; Fisher’s exact, two-sided, *P* < 0.05). We also intersected our cell-type-specific peak sets and predicted enhancers with de novo non-coding mutations (DNMs) identified from individuals with ASD and NDD, but no peak sets were significantly enriched for the currently annotated DNMs in probands compared to sibling controls (Extended Data Fig. 6d, e). In addition, we intersected predicted enhancers with topological associated domains (TADs) that contain genes associated with neurodevelopmental diseases^24,31^, and found significant colocalization in TADs in several cell types (Extended Data Fig. 6g; Fisher’s exact, two-sided, *P* < 0.05).

Finally, we sought to assess the enrichment of common variants associated with neuropsychiatric disease risk in our predicted enhancers for each cell type. To do this, we performed a partitioned heritability linkage disequilibrium (LD) score regression analysis using summary statistics from large-scale genome-wide association studies of schizophrenia^27,32^, ASD^33^, major depressive disorder^34^, and bipolar disorder^35^ (Methods). We found that excitatory and inhibitory neuron putative enhancers were enriched (FDR <0.05) for common variants associated with schizophrenia, confirming previous findings of neuronal involvement^27^ (Fig. 1k). Together, our prenatal cell-type-specific chromatin state data have the potential to identify specific regulatory programs during cortical development that confer the greatest risk for neurodevelopmental disorders, particularly as improved disease-associated variant annotations become available.

## Dynamic chromatin states in neurogenesis

**Fig. 2:**
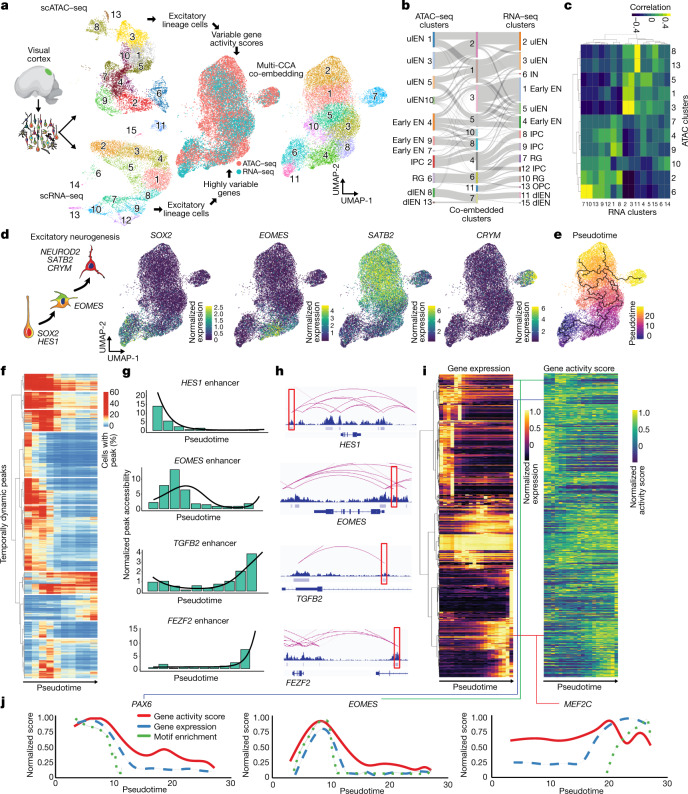
Dynamic changes in chromatin accessibility during human cortical neurogenesis. **a**, Workflow for co-embedding scATAC-seq and scRNA-seq data from the same samples. Left, experimental workflow. Top middle left, UMAP projection of scATAC-seq cells from visual cortex (*n* = 3 individuals) coloured by leiden clusters. Bottom middle left, UMAP projection of scRNA-seq cells from visual cortex (*n* = 2 individuals) colored by Leiden clusters. Middle right, UMAP projection of co-embedded cells coloured by assay. Right, UMAP projection of co-embedded scATAC-seq and scRNA-seq cells coloured by Leiden clusters. **b**, Sankey plot depicting mappings between scATAC-seq clusters, scRNA-seq clusters, and co-embedded clusters. **c**, Heat map of correlations between scATAC-seq and scRNA-seq clusters based on a set of cell-type marker genes (Methods). **d**, Left, schematic depicting cell-type marker genes in the cortical excitatory neuronal lineage. Right, projection of log-normalized gene expression and gene activity scores in co-embedded space for *SOX2* (RGs), *EOMES* (IPCs), *SATB2* (ulENs), and *CRYM* (dlENs). **e**, UMAP projection of co-embedded cells coloured by pseudotime with principal graph overlaid. **f**, Heat map depicting the average proportion of cells with peaks that are differentially accessible across pseudotime (*n* = 25,415). Cells are binned by pseudotime into ten equally sized bins. **g**, Peak accessibility for four individual peaks across ten pseudotime bins with regression line overlaid. **h**, Predicted enhancer–gene interactions (pink curves) overlaying ATAC-seq signal tracks and peaks with each of the four enhancers in **g** highlighted in red. **i**, Heat maps depicting gene expression (left) and gene activity scores derived from open chromatin (right) for 615 cell-type marker genes. Values are averaged within 20 equally sized bins of pseudotime. **j**, Comparison of moving averages of normalized gene activity scores (red), gene expression (blue), and motif enrichment (green) across pseudotime for *PAX6* (left), *EOMES* (middle), and *MEF2C* (right).

To better understand how transcriptomic and epigenomic changes may regulate cell fate decisions during neurogenesis, we co-embedded scRNA-seq and scATAC-seq datasets for the relevant cell types generated from the visual cortex (Fig. 2a–c, Methods). Projections of gene expression and gene activity scores in the co-embedded space revealed that clustering of distinct cell types is preserved irrespective of the profiling modality (Fig. 2d). To identify the trajectories of chromatin accessibility that underlie the differentiation and maturation of excitatory neurons, we performed pseudotemporal ordering of cells in the co-embedded space, which recovered the known developmental sequence of cell types undergoing excitatory neuron differentiation (Fig. 2e,  Methods). We identified more than 25,000 peaks with transient accessibility across pseudotime, including more than 5,000 predicted enhancers, many of which are predicted to interact with genes linked to cell type identity (Fig. 2f–h, Supplementary Table 9).

**Extended Data Fig. 7:**
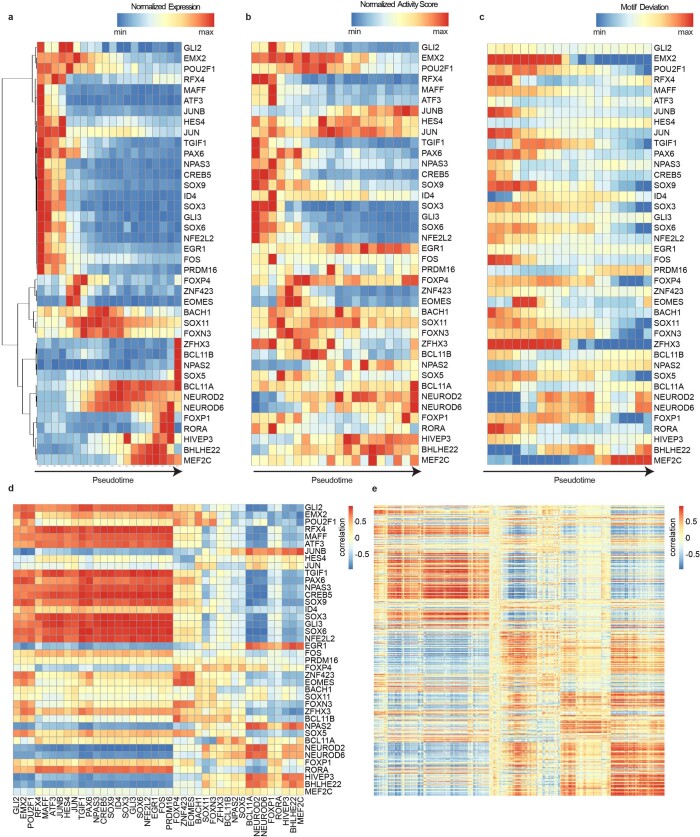
Dynamic patterns of gene expression, chromatin accessibility, and transcription factor motif enrichment across pseudotime. **a**–**c**, Heat map of normalized expression (**a**), normalized gene activity scores (**b**) and deviation scores of motif enrichments (**c**) of 40 key lineage transcription factors across pseudotime. Pseudotime is binned into 20 equally sized bins and expression, activity scores and deviations are averaged across all cells in each bin. Deviation scores determined using ChromVAR^26^ (Methods). **d**, **e**, Heat maps of correlations between gene expression and gene activity scores for 40 key lineage transcription factors (**d**) and for 615 cell-type marker genes (**e**).

Consistent with recent reports^36,37^, for genes with variable expression across pseudotime, gene activity scores derived from chromatin accessibility in the *cis*-regulatory region around genes were highly correlated with gene expression (Fig. 2i, Extended Data Fig. 7, Methods). Finally, by calculating transcription factor binding site enrichment across peaks that showed dynamic changes in accessibility along pseudotime, we reconstructed the known hierarchy of transcription factors involved in cortical neurogenesis, including sequential enrichment for PAX6, EOMES, and MEF2C binding sites among transiently accessible loci (Fig. 2j). Together, these results underscore the highly dynamic states of chromatin accessibility during human cortical neurogenesis.

## Area-specific chromatin states

**Fig. 3:**
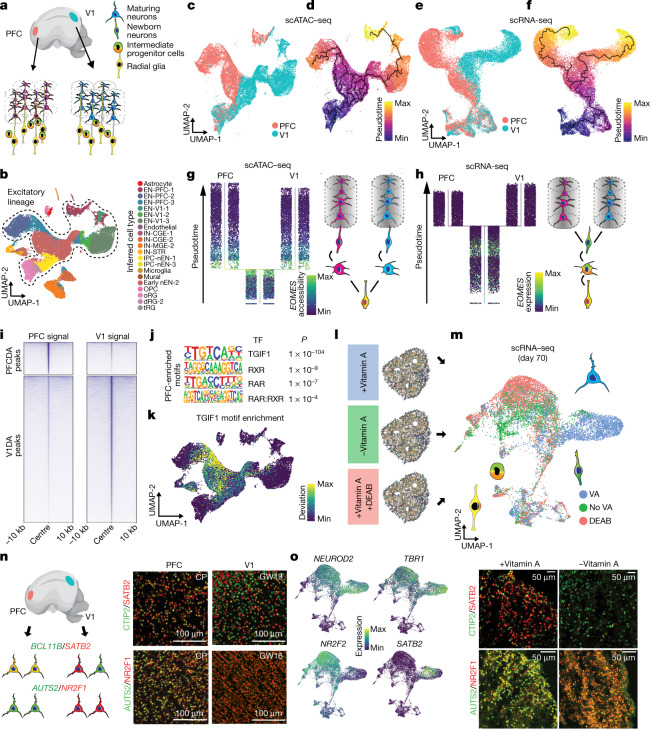
Areal differences in chromatin state of progenitor cells foreshadow the emergence of area-specific types of excitatory neurons. **a**, Differentiation trajectories for excitatory neurons from the PFC (left) and V1 (right). **b**, UMAP projection of PFC and V1 scATAC-seq cells (*n* = 3 individuals) coloured by cell type predictions. Cells from the excitatory lineage are outlined. IN-STR, striatal interneurons. Suffixes 1–3 denote subclusters from Nowakowski et al.^2^. **c**, **d**, UMAP projections of PFC and V1 scATAC-seq excitatory lineage cells coloured by area of origin (**c**) and pseudotime value (**d**). **e**, **f**, UMAP projections of PFC and V1 scRNA-seq excitatory lineage cells (*n* = 2 individuals) coloured by area of origin (**e**) and pseudotime value (**f**). **g**, **h**, Left, PFC and V1 scATAC-seq (**g**) and scRNA-seq (**h**) excitatory lineage cells ordered from bottom to top by pseudotime value with PFC–V1 divergence branch point shown (Methods). Cells coloured by *EOMES* gene activity score (**g**) or expression (**h**), highlighting IPCs. Right, schematic illustrating the excitatory neuron differentiation trajectory based on chromatin accessibility, in which PFC–V1 divergence becomes apparent at the level of IPCs (**g**), or gene expression, in which PFC–V1 divergence is not apparent in IPCs (**h**). **i**, Pile-ups of PFC and V1 signal in PFC and V1 differentially accessible (DA) peak sets. Pileups are centred on peaks and show ±10-kb flanking regions. **j**, Transcription factor motif enrichments of RA-related transcription factors in set of 4,176 PFC-specific peaks (Fisher’s exact, two-sided, FDR < 0.05). **k**, UMAP projection of deviation scores of motif enrichment for TGIF1. **l**, Experimental design to test role of RA in organoid area identity. **m**, UMAP projection of scRNA-seq data from day 70 organoids (*n* = 11,415 cells). Cells coloured by treatment. VA, vitamin A. **n**, Left, schematic of expected expression patterns of *BCL11B*, *SATB2*, *AUTS2*, and *NR2F1* in primary human cortex. Right, images of primary developing human cortex from the PFC (left) and V1 (right) immunostained for CTIP2 and SATB2 (top) or AUTS2 and NR2F1 (bottom). Representative images shown from *n* = 2 specimens. **o**, Left, UMAP projection of cells coloured by expression of *NEUROD2*, *TBR1*, *SATB2*, and *NR2F2*. Right, images of organoids cultured without (left) or with vitamin A (right) immunostained for CTIP2 and SATB2 (top) or AUTS2 and NR2F1 (bottom). Representative images shown from *n* = 3 lines.

**Extended Data Fig. 8:**
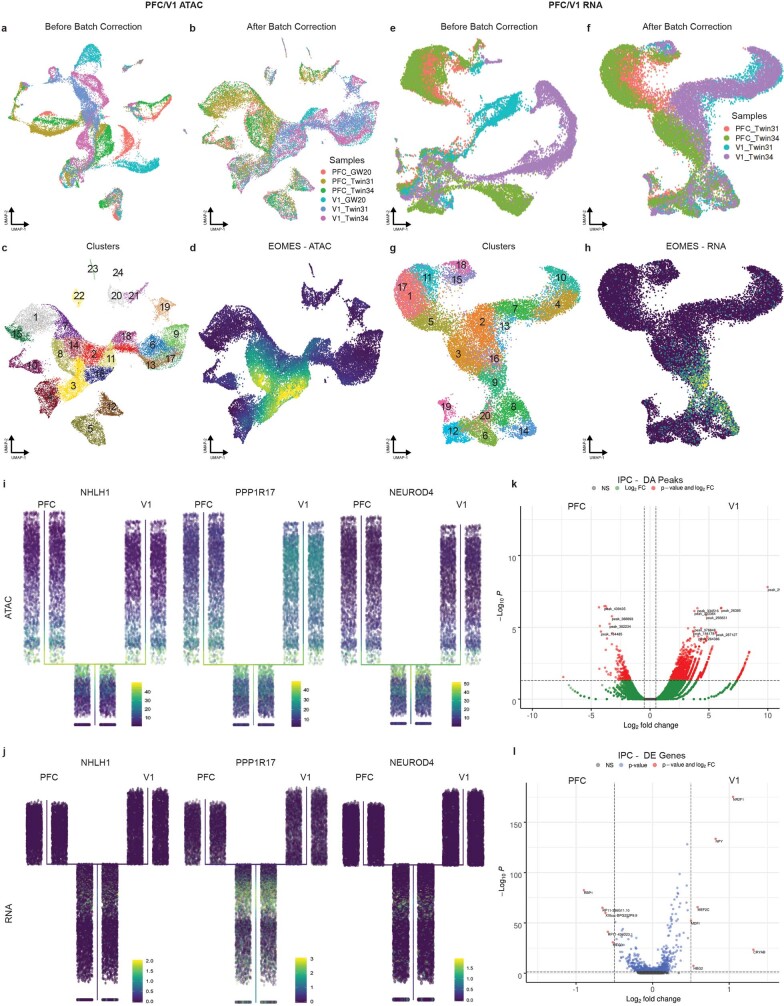
Chromatin state profiling reveals divergence of PFC and V1 excitatory lineages. **a**–**d**, UMAP projections of scATAC-seq cells from PFC and V1 samples before batch correction coloured by sample (**a**), after batch correction coloured by sample (**b**), coloured by Leiden cluster (**c**) and coloured by gene activity score for *EOMES*, a marker of IPCs (**d**). **e**–**h**, UMAP projections of scRNA-seq cells from PFC and V1 samples before batch correction coloured by sample (**e**), after batch correction coloured by sample (**f**), coloured by Leiden cluster (**g**) and coloured by gene activity score for *EOMES*, a marker of IPCs (**h**). **i**, **j**, Projection of *NHLH1*, *PPP1R17*, and *NEUROD4* gene activity scores on PFC and V1 scATAC-seq cells (**i**) and scRNA-seq cells (**j**) ordered by pseudotime with PFC/V1 divergence branch points displayed. **k**, Volcano plot of peaks that are differentially accessible between PFC and V1 IPCs. Peaks highlighted in red have log-transformed fold change (logFC) > 0.5 and FDR < 0.05 (*n* = 1,819). **l**, Volcano plot of genes that are differentially expressed between PFC and V1 IPCs. Genes highlighted in red have logFC > 0.5 and FDR < 0.05 (*n* = 11).

Area-specific types of cortical excitatory neurons emerge during early neurogenesis, but only limited transcriptomic differences have been found among progenitors from different regions^2^. Given that changes in the accessibility of regulatory elements often precede gene expression, we investigated whether epigenomic signatures could foreshadow the emergence of area-specific excitatory neurons. Specifically, we compared scRNA-seq and scATAC-seq profiles of excitatory lineage cells sampled from the extremes of the rostral–caudal axis, PFC and V1 (Fig. 3a, b, Extended Data Fig. 8a–h). For each modality, we ordered the cells in pseudotime to approximate the differentiation trajectory and identified the ‘branch’ point along this trajectory at which transcriptomic or chromatin state differences between PFC and V1 lineages become apparent (Fig. 3c–f, Methods). In contrast to transcriptomic data, which have revealed area-specific clusters of excitatory neurons (Fig. 3h), chromatin state signatures revealed a notable divergence between PFC and V1 intermediate progenitor populations (Fig. 3g, Extended Data Fig. 8i, j). Transcriptomically, PFC and V1 IPCs differentially expressed only a handful of genes, including *NR2F1* (Supplementary Table 24), whereas chromatin accessibility analysis identified more than 1,800 differentially accessible peaks between these cell types (Extended Data Fig. 8k, l, Supplementary Table 25).

**Extended Data Fig. 9:**
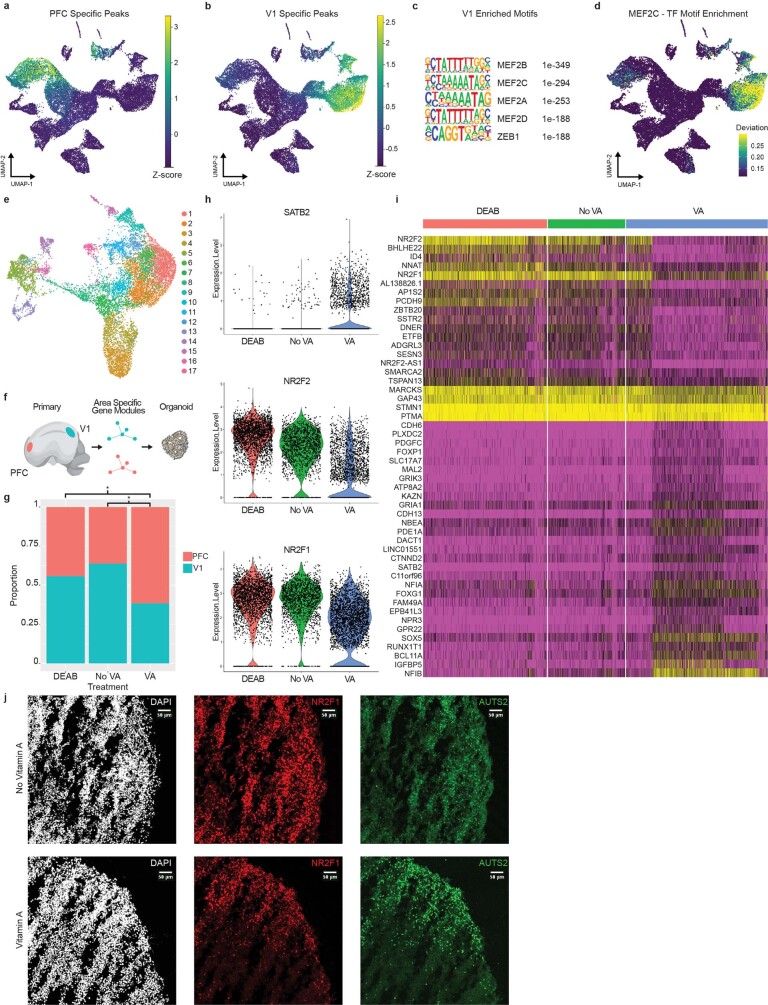
Modelling the PFC–V1 split in the developing cortex. **a**, **b**, UMAP projections of *Z*-scores of enrichment of PFC-specific peaks (**a**; *n* = 4,176) and V1-specific peaks (**b**; *n* = 21,030) in all PFC and V1 scATAC-seq cells (Fisher’s exact, two-sided, FDR < 0.05). **c**, Top enriched transcription factor motifs in V1-specific peak set as determined by HOMER (hypergeometric test, one-sided). **d**, UMAP projection of ChromVAR deviation scores of motif enrichment of MEF2C in all PFC and V1 scATAC-seq cells. **e**, UMAP projection of scRNA-seq data from organoids (*n* = 3) cultured in the presence of vitamin A, without the presence of vitamin A, and in the presence of DEAB. Cells coloured by cluster. **f**, Schematic depiction of classifier method used to assign area identity to organoid cells on the basis of defined area-specific gene modules. **g**, Bar plot depicting proportion of excitatory neurons from each treatment group classified as more PFC-like or more V1-like on the basis of calculation of module eigengene values for area-specific modules (see Methods). Asterisks indicate significant differences in proportions (DEAB: PFC, 1,160/2,622; V1, 1,462/2,622; NoVA: PFC, 563/1,556; V1, 993/1,556; VA: PFC, 1,831/2,976; V1, 1,145/2,976) (*χ*^2^ test, one-sided, vitamin A versus no vitamin A: *P* = 3.209 × 10^–59^; vitamin A versus DEAB: *P* = 2.79 × 10^−38^). **h**, Violin plots depicting expression levels of SATB2, NR2F1, and NR2F2 for excitatory neurons from each treatment group. **i**, Heat map showing gene expression of differentially expressed genes between excitatory neurons cultured with and without vitamin A. **j**, Images of organoids cultured with and without vitamin A stained with DAPI and immunostained for NR2F1 and AUTS2. All images taken at 10× resolution. Representative images shown from *n* = 3 lines.

Next, to identify putative regulatory programs that could underlie the divergence of the PFC and V1 lineages, we performed transcription factor binding site enrichment analysis on peaks that were differentially accessible between PFC and V1 cells (Fisher’s exact test, two-sided, FDR < 0.05; Fig. 3i–k, Extended Data Fig. 9a–d, Supplementary Tables 10, 11). This analysis identified several transcription factors that had been predicted on the basis of transcriptomic studies^2,38^, including enrichment of POU3F2, MEIS1, TBR1, NEUROD1, NEUROG2, and TBX21 binding motifs among PFC cells. Notably, this analysis also identified components of the retinoic acid (RA) signalling pathway, including RXR, RAR and TGIF1, among PFC cells, consistent with recent evidence that RA activity is increased in the PFC during mid-gestation^39^ (Fig. 3j, k, Supplementary Table 21).

## Retinoic acid in cortical arealization

Retinoic acid signalling has an important role in patterning of the neural tissue during mammalian brain development^39,40^. To test whether RA promotes the differentiation of human PFC lineages, we cultured cortical organoids in the presence or absence of vitamin A (the precursor for RA synthesis). In parallel, we treated organoids that were cultured with vitamin A with 4-diethylamniobenzaldehyde (DEAB), a potent inhibitor of RA synthesis^41^ (Methods, Fig. 3l). At week 10 of differentiation, which corresponds to deep layer neurogenesis, we profiled organoids using scRNA-seq. We found that excitatory forebrain neurons (*FOXG1/NEUROD2* double-positive) cultured in the presence of vitamin A clustered separately from those derived from organoids cultured without vitamin A or in the presence of DEAB (Fig. 3m, n, Extended Data Fig. 9e). Among the top differentially expressed genes, we found signatures that distinguished PFC and V1 cortical neurons, including *SATB2*, *NR2F1*, and *NR2F2*^2^ (Fig. 3n, Extended Data Fig. 9h, i). We applied a previously developed classifier for annotating PFC and V1 neuronal identities among organoid neurons^42^, and found consistently higher proportion of neurons classified as PFC-like among organoids cultured with vitamin A compared with those cultured without vitamin A or treated with DEAB (Extended Data Fig. 9f, g; *χ*^2^ test, one-sided, *P* < 0.00001). The differential expression of gene products, including co-expression among excitatory neurons of SATB2, CTIP2 and AUTS2 in prefrontal cortex and enriched expression of NR2F1 in the visual cortex^2,43–45^, was confirmed by immunostaining and found to be consistent with a PFC-like identity of organoids cultured in the presence of vitamin A (Fig. 3n, o, Extended Data Fig. 9j). Together, these findings suggest that the RA signalling pathway contributes to the specification of the PFC neuronal lineage during human cortical development; further studies are required to determine how the RA pathway interfaces with other signalling pathways, such as the fibroblast growth factor pathway, to promote this neuronal fate^46^.

## Benchmarking cerebral organoids

**Fig. 4:**
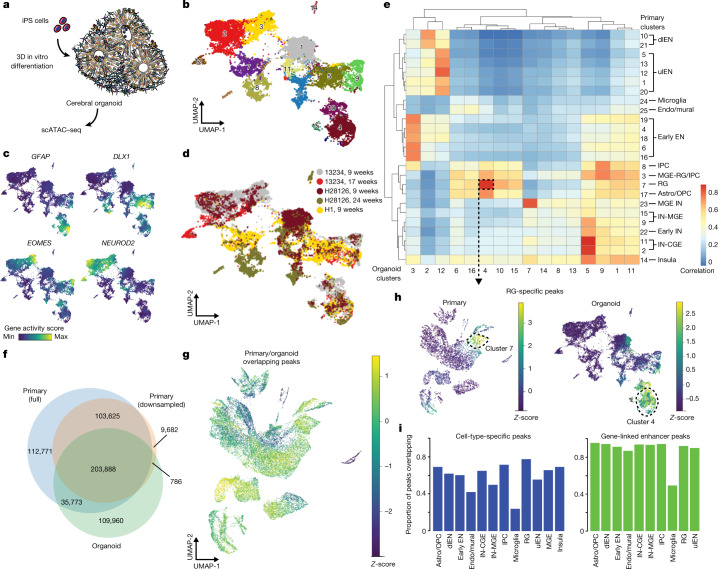
Cell type-specific differences in chromatin accessibility between cerebral organoids and the developing human brain. **a**, Schematic depicting experimental workflow. iPS cells, induced pluripotent stem cells. **b**, UMAP projection of all organoid scATAC-seq cells (*n* = 5 organoids from 3 different lines and 3 different time points; 23,555 cells) coloured by Leiden clusters. (Cluster 16 not depicted, see Extended Data Fig. 10e.) **c**, UMAP projections of gene activity scores for *GFAP* marking RGs, *EOMES* marking IPCs, *DLX1* marking interneurons, and *NEUROD2* marking excitatory neurons. **d**, UMAP projection of all organoid scATAC-seq cells coloured by sample. **e**, Heat map of Pearson correlations between primary and organoid scATAC-seq clusters based on a common peak set. MGE IN, interneurons identified in the MGE. **f**, Venn diagram of overlap between the full primary peak set (red), a down-sampled primary peak set (blue), and the organoid peak set. **g**, UMAP projection of enrichment *Z*-scores of peaks that overlap between primary and organoid datasets on the primary scATAC-seq dataset. **h**, UMAP projections of enrichment *Z*-scores of RG-specific peaks (Fisher’s exact, two-sided, FDR < 0.05) in all primary scATAC-seq cells (left) and all organoid scATAC-seq cells (right). **i**, Left, proportion of cell-type-specific primary peaks in the organoid peak set. Right, proportion of gene-linked enhancers for each cell type in the organoid peak set.

**Extended Data Fig. 10:**
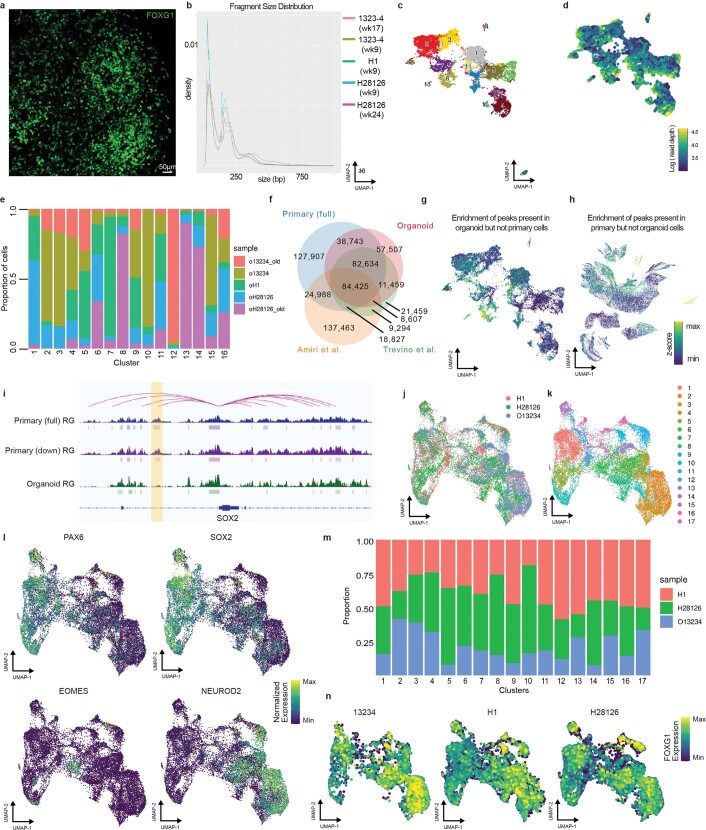
Comparison of organoid and primary peaks reveal significant differences in chromatin landscapes. **a**, Representative image of organoid (1323-4 line depicted) immunostained for FOXG1, a marker of cortical identity. Image taken at 10× resolution. Representative image shown from *n* = 3 lines. **b**, Density curves of fragment size distributions for each organoid sample. Fragments are deduplicated, uniquely mapped fragments that have passed quality filters (see Methods). **c**, UMAP projection of all organoid scATAC-seq cells coloured by cluster. **d**, UMAP projection of log(read depth). Fragments are deduplicated, uniquely mapped fragments that have passed quality filters (see Methods). **e**, Bar plot depicting the proportions of cells in each cluster from each organoid sample. **f**, Venn diagram of overlaps of all primary scATAC-seq peaks, all organoid scATAC–seq peaks, all H3K27ac peaks from Amiri et al.^11^, and all ATAC-seq peaks from Trevino et al.^49^. **g**, UMAP projection of *Z*-scores of enrichment of peaks that are present in the organoid scATAC-seq dataset but not in the primary scATAC-seq dataset. **h**, UMAP projection of *Z*-scores of enrichment of peaks that are present in the primary scATAC-seq dataset but not in the organoid scATAC-seq dataset. **i**, Genome browser tracks depicted a predicted enhancer of *SOX2* that is present in both the full and down-sampled primary RGs but not detected in organoid RGs. **j**, UMAP projection of scRNA-seq data from the same three organoid lines used for scATAC-seq analysis coloured by line (*n* = 19,509 cells). **k**, UMAP projection of scRNA-seq data coloured by cluster. **l**, UMAP projection of normalized gene expression of *PAX6*, *SOX2*, *EOMES*, and *NEUROD2*. Maximum value was set at the 99th quantile. **m**, Bar plot depicting the proportions of cells in each cluster from each organoid sample. **n**, UMAP projection of normalized gene expression of *FOXG1* split by organoid line. Maximum value was set at the 99th quantile.

Owing to the scarcity of primary human tissue, studies of human neural development require suitable in vitro models, such as cerebral organoids. Previous studies have emphasized the similarities between cerebral organoid cells and their in vivo counterparts using single-cell transcriptomics^47,48^ and bulk epigenomics^10,11,49^. We generated scATAC-seq data for 23,555 cells from cortical organoids derived by directed differentiation from three genetically normal individuals^47,50^ at three time points of differentiation (Fig. 4a–c, Extended Data Fig. 10a–e, Supplementary Table 1, Methods). To validate our organoid lines, we also generated scRNA-seq data from organoids derived from the same lines and cultured in parallel and showed that all lines expressed *FOXG1* and markers of major cell types (Extended Data Fig. 10j–n). Using gene activity scores, we identified the major classes of cell types among scATAC-seq data, including RGs, IPCs, interneurons, and excitatory neurons, although individual clusters contained fewer cell-type-specific peaks than clusters derived from primary cells (Fig. 4d, Supplementary Table 17).

Next, we quantified chromatin accessibility among organoid cells across peaks defined from primary cells (Methods, Fig. 4e) and found organoid cells homologous to RGs, IPCs, interneurons, and excitatory neurons. Across organoid cells, we identified 377,448 peaks (Supplementary Table 16), and intersected them with the primary cell peaks, as well as with a down-sampled set to match the cell abundance of the organoid dataset (Methods, Fig. 4f). Of the 459,953 peaks in the full primary dataset, 239,661 were also called in the organoid dataset (Fig. 4g, Extended Data Fig. 10f, Supplementary Table 18). The set of peaks not detected in organoids was strongly enriched for peaks specific to cell types not found in substantial numbers in our organoids, including microglia, endothelial cells, astrocytes, and OPCs (Extended Data Fig. 10h). After we removed these peaks from the analysis, the remaining peaks not found in organoids were enriched among V1 excitatory neurons. While cell-type-specific peaks identified in primary cells maintained cell-type specificity in homologous organoid cell types, many of the cell-type-specific peaks were not detected (Fig. 4h, i). However, the majority (more than 80%) of predicted enhancers were also identified in organoids, except for microglial enhancers (Fig. 4i, Extended Data Fig. 10h). We also identified 109,960 organoid peaks that were not found in the primary cell dataset (Fig. 4f, Extended Data Fig. 10g). Transcription factor motif enrichment analysis revealed that these organoid-specific peaks were enriched for HIF1A, HIF1B, and p53, which is consistent with the reported higher levels of cellular stress in organoids^42^ (Supplementary Table 23). To further explore the robustness of our findings, we integrated our data with published epigenomic datasets generated from organoids^11,49^. This integrative analysis revealed that 20,066 out of the 77,573 peaks found in organoids, but not primary tissue, were also detected in other published datasets (Extended Data Fig. 10f, Supplementary Table 19), suggesting that our findings can be independently validated.

## Discussion

In this study, we profiled the chromatin state of single cells in the developing human brain and found thousands of transiently accessible loci that track with neuronal differentiation. These states may reveal mechanisms that govern the establishment of cell fate during neurogenesis, and intersecting them with comparable datasets from the adult human brain may enable the complete reconstruction of the epigenomic neurodevelopmental trajectory^51^. Consistent with previous studies^52^, intersection of the chromatin state landscape with disease variants implicated post-mitotic, developing cortical excitatory neurons in the aetiopathogenesis of neuropsychiatric disorders^9,22,53^. Future studies are needed to probe how disease-associated variants in these regulatory regions modify cell fate decisions in the developing cortex. By comparing the regulatory landscapes of different cortical areas during development (V1 and PFC), we found distinct sets of transcription factor binding motifs that were differentially enriched between these two lineages. Our findings extend the established role of RA signalling in forebrain development^39,45^ and suggest that RA signalling contributes to the specification of excitatory neurons of the human PFC. Dysregulation of RA signalling has been implicated in a range of neurodevelopmental and psychiatric disorders^54^, and therefore our findings may have implications for studies of these disorders.

## Methods

### Tissue source

De-identified tissue samples were collected with previous patient consent in strict observance of the legal and institutional ethical regulations. Protocols were approved by the Human Gamete, Embryo, and Stem Cell Research Committee (institutional review board) at the University of California, San Francisco.

### Nucleus isolation from fresh primary tissue

Cortical areas were microdissected from three specimens of mid-gestation human cortex, in addition to three specimens of non-area-specific mid-gestation human cortex. Tissue was dissociated in Papain containing DNase I (DNase) for 30 min at 37 °C and samples were triturated to form a single-cell suspension. Cells (10^6^) were pelleted and lysed for 3 min in 100 μl chilled lysis buffer (10 mM Tris-HCl pH 7.4, 10 mM NaCl, 3 mM MgCl_2_, 0.1% Tween-20, 0.1% Igepal CA-630, 0.01% digitonin, 1% bovine serum albumin (BSA)). Lysed cells were then washed with 1 ml chilled wash buffer (10 mM Tris-HCl pH 7.4, 10 mM NaCl, 3 mM MgCl_2_, 0.1% Tween-20, 1% BSA) and nuclei were pelleted at 500g for 5 min at 4 °C.

### Nucleus isolation from frozen primary tissue

Tissue sections were snap frozen and stored at −80 °C. Nuclei were isolated from frozen tissues as described^57^. In brief, frozen tissue samples were thawed in 2 ml chilled homogenization buffer (10 mM Tris pH 7.8, 5 mM CaCl_2_, 3 mM magnesium acetate, 320 mM sucrose, 0.1 mM EDTA, 0.1% NP40, 167 μM β-mercaptoethanol, 16.7 μM PMSF) and lysed in a pre-chilled dounce. Cell lysates were then centrifuged in an iodixanol gradient for 20 min at 3,000g at 4 °C in a swinging bucket centrifuge with the brake turned off. The nucleus band was then carefully pipetted and nuclei were diluted in wash buffer.

### Cortical organoid differentiation

Cortical organoids were cultured using a forebrain-directed differentiation protocol^47,58^. In brief, two genetically normal human iPS cell lines (H28126 (Gilad Laboratory, University of Chicago) and 1323-4 (Conklin Laboratory, Gladstone Institutes)), which were previously authenticated^47^, and the embryonic stem cell line H1 (WiCell, authenticated at source), were expanded and dissociated into single cells using accutase. Cells tested negative for mycoplasma. After dissociation, cells were reconstituted in neural induction medium at a density of 10,000 cells per well in 96-well v-bottom low-adhesion plates. Glasgow’s modified Eagle’s medium (GMEM)-based neural induction medium includes 20% Knockout Serum Replacer (KSR), 1× non-essential amino acids, 0.11 mg/ml sodium pyruvate, 1× penicillin-streptomycin, 0.1 mM β-mercaptoethanol, 5 μM SB431542 and 3 μM IWR1-endo. Medium was supplemented with 20 μM Rock inhibitor Y-27632 for the first 6 days. After 18 days, organoids were transferred from 96- to 6-well low-adhesion plates, moved to an orbital shaker rotating at 90 rpm, and changed to Dulbecco’s modified Eagle’s medium (DMEM)/F12-based medium containing 1× glutamax, 1× N2, 1× B27 without vitamin A and 1× antibiotic–antimycotic (anti-anti). At 35 days, organoids were moved into DMEM/F12-based medium containing 1× N2, 1× B27 with vitamin A and 1× anti-anti. Throughout the culture duration, organoids were fed every other day.

### Nucleus isolation from cerebral organoids

Cerebral organoids were dissociated in papain containing DNase I (DNase) for 30 min at 37 °C and samples were triturated to form a single-cell suspension. Cells (10^6^) were pelleted and lysed for 3 min in 100 μl chilled lysis buffer (10 mM Tris-HCl pH 7.4, 10 mM NaCl, 3 mM MgCl_2_, 0.1% Tween-20, 0.1% Igepal CA-630, 0.01% digitonin, 1% BSA). Lysed cells were then washed with 1 ml chilled wash buffer (10 mM Tris-HCl pH 7.4, 10 mM NaCl, 3 mM MgCl_2_, 0.1% Tween-20, 1% BSA) and nuclei were pelleted at 500g for 5 min at 4 °C.

### Cortical organoid arealization experiment

Two genetically normal iPS cell lines (1323-4 and H28126) were differentiated into cortical organoids following the above protocol up to day 35. At day 35, organoids from each line were split into three different conditions: 1) normal medium conditions for day 35 and beyond as described above (with vitamin A); 2) normal medium conditions for day 35 and beyond (with vitamin A) plus 100 μM DEAB, an inhibitor of RA synthesis; or 3) normal medium conditions for day 35 and beyond as described above except using B27 without vitamin A. DEAB treatment was ended after one week, and culture conditions remained otherwise the same until day 70, at which time organoids were processed for scRNA-seq and fixed for immunohistochemistry. We used 1323-4 organoids for scRNA-seq (one for each of the three conditions) and both 1323-4 and H28126 organoids were used for immunostaining. Organoids processed for scRNA-seq were multiplexed using multi-seq oligonucleotide barcoding^59^ and pooled for library preparation and sequencing to reduce potential batch effects.

### Single-cell RNA-seq library preparation and sequencing

Single-cell RNA-seq libraries were generated using the 10X Genomics Chromium 3′ Gene Expression Kit. In brief, single cells were loaded onto chromium chips with a capture target of 10,000 cells per sample. Libraries were prepared following the provided protocol and sequenced on an Illumina NovaSeq with a targeted sequencing depth of 50,000 reads per cell. BCL files from sequencing were then used as inputs to the 10X Genomics Cell Ranger pipeline.

### Bulk ATAC-seq library preparation and sequencing

Bulk ATAC-seq libraries were generated as described^57^. In brief, 50,000 nuclei were permeablized and tagmented. Tagmented chromatin libraries were generated and sequenced on an Illumina NovaSeq with a target sequencing depth of 50 million reads per library. Sequencing data were used as an input to the ENCODE ATAC-seq analysis pipeline (https://github.com/ENCODE-DCC/atac-seq-pipeline).

### Bulk H3K27ac CUT&Tag library preparation and sequencing

H3K27ac CUT&Tag libraries were prepared as previously described^60^, with modifications. In brief, cells were dissociated from human developing cortical tissue as described above. Fifty thousand cell aliquots were pelleted at 600*g* in a swinging bucket rotor centrifuge and washed twice in 200 μl CUT&Tag wash buffer (20 mM HEPES pH 7.5, 150 mM NaCl, 0.5 mM spermidine, 1× protease inhibitor cocktail (Roche)). Nuclei were isolated by resuspending cell pellets in 200 μl dig-wash buffer (CUT&Tag wash buffer supplemented with 0.05% digitonin and 0.05% IGEPAL CA-630). Nucleus pellets were washed twice in 200 μl dig-wash buffer before resuspension in 100 μl dig-wash buffer supplemented with 2 mM EDTA and a 1:50 dilution of H3K27ac primary antibody (Cell Signaling 8173), and incubated overnight at 4 °C on an overhead rotator. Excess primary antibody was removed by pelleting the nuclei at 600*g* and washing twice in 200 μl dig-wash buffer. Secondary antibody (Novex A16031) was added at a dilution of 1:50 in 100 μl dig-wash buffer and nuclei were incubated at room temperature for 30 min while rotating. Excess secondary antibody was removed by pelleting the nuclei at 600*g* and washing twice in 200 μl dig-wash buffer. pA-Tn5 was added at a dilution of 1:100 in 100 μl dig-med buffer (0.05% digitonin, 20 mM HEPES, pH 7.5, 300 mM NaCl, 0.5 mM spermidine, 1× protease inhibitor cocktail), and nuclei were incubated at room temperature for 1 h while rotating. Unbound pA-Tn5 was removed by pelleting the nuclei at 300*g* and washing twice in 200 μl dig-med buffer. Nuclei were resuspended in 100 μl tagmentation buffer (10 mM MgCl_2_ in dig-med buffer) and incubated for 1 h at 37 °C. After tagmentation, nuclei were lysed with the addition of 100 μl DNA binding buffer (Zymo Research), and tagmented DNA was purified with a 1.5:1 ratio of AMPure XP beads (Beckman) following the manufacturer’s instructions. Purified DNA was eluted in 21 μl buffer EB (10 mM Tris-Cl, pH 8.5) and mixed with 2 μl each 10 μM indexed i5 and i7 primers and 25 μl NEBNext HiFi 2 × PCR Master mix. Libraries were amplified with the cycling conditions: 72 °C for 5 min; 98 °C for 30 s; 12 cycles of 98 °C for 10 s and 63 °C for 30 s; final extension at 72 °C for 1 min and hold at 4 °C. Libraries were purified with a 1:1 ratio of AMPure XP beads and eluted in 15 μl EB. CUT&Tag libraries were quantified by Agilent Bioanalyzer, and sequenced paired-end to a depth of 15 million reads on an Illumina NovaSeq 6000 system, with read lengths 50 × 8 × 8 × 50.

### Single-cell ATAC-seq library preparation and sequencing

Nuclei were prepared as outlined in the 10X Genomics Chromium single-cell ATAC-seq solution protocol (v1.0 kit was used). Nuclei were loaded with a capture target of 10,000 nuclei per sample. scATAC-seq libraries were prepared for sequencing following the 10X Genomics single-cell ATAC–seq solution protocol. scATAC-seq libraries were sequenced using PE150 sequencing on an Illumina NovaSeq with a target depth of 25,000 reads per nucleus (Supplementary Table 1).

### Single-cell ATAC-seq analysis pipeline

#### Cell Ranger

BCL files generated from sequencing were used as inputs to the 10X Genomics Cell Ranger ATAC pipeline. In brief, FASTQ files were generated and aligned to GRCh38 using BWA. Fragment files were generated containing all unique properly paired and aligned fragments with mapping quality (MAPQ) >30. Each unique fragment was associated with a single cell barcode.

#### SnapATAC

Fragment files generated from the Cell Ranger ATAC pipeline were loaded into the SnapATAC^61^ pipeline (https://github.com/r3fang/SnapATAC) and Snap files were generated. A cell-by-bin matrix was then generated for each sample by segmenting the genome into 5-kb windows and scoring each cell for reads in each window. Cells were filtered based on log(reads passed filters) between 3 and 5 and fraction of reads in promoters between 10 and 60% to obtain cells with high quality libraries. Bins were then filtered, removing bins overlapping ENCODE blacklist regions (http://mitra.stanford.edu/kundaje/akundaje/release/blacklists/). This matrix was then binarized and coverage of each bin was calculated and normalized by log_10_(count + 1). *Z*-scores were calculated from normalized bin coverages and bins with a *Z*-score beyond ± 2 were filtered from further analysis. Cells with coverage of <500 bins were removed from the downstream analysis. A cell-by-cell similarity matrix was generated by calculating the latent semantic index (LSI) of the binarized bin matrix. Singular value decomposition (SVD) was performed on the log (term frequency – inverse document frequency) (TF − IDF) matrix. The top 50 reduced dimensions were used for batch correction through scAlign.

#### scAlign batch correction

Multiple batches were integrated using the scAlign package^16^ (https://github.com/quon-titative-biology/scAlign). The ATAC batches were first merged together to calculate the latent semantic index (LSI) with the transcription factor matrix log-scaled for input into SVD. The 50 reduced dimensions of LSI were used as inputs to the encoder. The latent dimension was set at 32 and ran with all-pairs alignment of all batches. The input dimension to the encoder was set to 50 to match the input dimensions and trained to 15,000 iterations using the small architecture setting with batch normalization. The 32 dimensions were used for downstream analysis for finding neighbours. The scRNA-seq were processed using Seurat and computed the top 15 components from CCA for input into scAlign, and the latent dimension was set to 20 using the small architecture with batch normalization and 15,000 iterations. All alignments were unsupervised.

#### Clustering and visualization

To visualize the high-dimensionality dataset in 2D space, the latent dimensions for the ATAC and RNA data from scAlign were used to construct UMAP^62^ graphs from Seurat. A *k*-nearest neighbour graph was constructed from the latent dimensions from scAlign using *k* = 15. The Leiden algorithm was then used to identify ‘communities’, or clusters, in the sample, representing groups of cells likely to be of the same cell type using resolution 0.8.

#### Calculating gene activity scores

To create a proxy for gene expression, ATACseq fragments in the gene body plus promoter (2 kb upstream from transcription start sites) of all protein-coding genes were summed for each cell to generate ‘gene activity scores’. A matrix was constructed for all gene activity scores by all cells. Owing to the sparsity of scATAC-seq data, the MAGIC^63^ imputation method was used, as implemented in the SnapATAC package, to impute gene activity scores based on the *k*-nearest neighbour graph.

#### Assigning cell type labels to scATAC-seq cells

Broad cell-type classes were assigned to cells on the basis of the gene activity scores of previously described cell-type marker genes^2^ (Extended Data Fig. 2a). To identify cell types at a higher resolution, we assigned cell-type labels using the CellWalker^17^ method, as implemented in CellWalkR (v0.1.7). In brief, we used CellWalker to integrate scRNA-seq derived labels^2^ with scATAC-seq data by building a network of label-to-cell and cell-to-cell edges and diffusing label information over this combined network to compensate for data sparsity in single-cell data. We calculated cell-to-cell edge weight using the Jaccard similarity between cells. Label-to-cell weight was calculated as the sum of the products of the gene activity scores for that cell and the log fold-change in expression of each marker for that cell label. We tuned label edge weight using cell homogeneity as described^17^. Diffusion resulted in a vector of influence scores of each label for each cell. We then smoothed these vectors for each cell by taking a weighted average of its scores with those of each of its ten closest neighbours (weighted such that each neighbour contributed one-fifth as much as the cell in question) in UMAP space. Finally, we assigned cell-type labels to each cell using the label with the highest influence.

#### Peak calling

Fragments from cells were grouped together by broad cell class (RG, IPC, ulEN, dlEN, endo/mural, astro/oligo, nEN, IN-MGE, IN-CGE, MGE progenitor, insular, microglia) and peaks were called on all cluster fragments using MACS2 (https://github.com/taoliu/MACS) with the parameters ‘--nomodel --shift -37 --ext 73 --qval 5e-2 -B --SPMR --call-summits’. Peaks from each cell type were then combined, merging overlapping peaks, to form a master peak set, and a cell-by-peak matrix was constructed. This matrix was binarized for all downstream applications.

#### Determination of differentially accessible peaks

Differentially accessible peaks for each cell type were determined by performing a two-sided Fisher’s exact test and selecting peaks that had log-transformed fold-change >0, and FDR-corrected *P* < 0.05, using the built in function in snapATAC ‘findDAR’.

#### Visualizing cluster signal in peaks

The deeptools suite^64^ (https://deeptools.readthedocs.io/en/develop/) was used to visualize pileups of cluster-specific ATAC-seq signal (output from MACS2) in DA peak sets.

#### Intersection with 25-chromatin-state model

To comprehensively categorize our peaks in genomic features genome-wide, we intersected our peak set with the 25-state model from the Roadmap Epigenomics Project^18^, specifically using the data generated from sample E081, which was a sample of developing human brain. Enrichment of peaks within annotated regions of the genome was calculated using the ratio between the (number of bases in state AND overlap feature)/(number of bases in genome) and the [(number of bases overlap feature)/(number of bases in genome) × (number of bases in state)/(number of bases in genome)] as previously described^18^.

#### Intersection with epigenomic datasets

We intersected our peak sets with several epigenomic datasets including ATAC-seq peaks from de la Torre-Ubieta et al.^9^ (GEO: GSE95023), ATAC-seq peaks from Markenscoff-Papadimitriou et al.^23^ (GEO: GSE149268), H3K4me3 PLAC–seq promoter-interacting regions, generated from ENs, INs, IPCs, and RGs sorted from samples of developing human cortex^21^ that were graciously provided by the author, H3K27ac peaks from Amiri et al.^11^ (taken from supplementary tables of publication), ATAC-seq peaks from Trevino et al.^49^ (GEO: GSE132403), H3K27ac peaks from Li et al.^22^ (obtained from http://development.psychencode.org), and high-confidence enhancer predictions from Wang et al.^24^ (obtained from http://resource.psychencode.org/). Any peaks not already mapped to hg38 were lifted over using the UCSC LiftOver tool. Overlaps between peak sets were determined using the ‘findOverlaps’ function in R.

#### Transcription factor motif enrichment analysis

The findMotifsGenome.pl tool from the HOMER suite^65^ (http://homer.ucsd.edu/homer/) was used to identify transcription factor motif enrichments in peak sets. The ChromVAR^26^ R package was used to identify transcription factor motif enrichments at the single-cell level in scATAC-seq data. In brief, the peak-by-cell matrix from the snap object was used as an input, filtering for peaks open in at least 10 cells. Biased-corrected transcription factor motif deviations were calculated for the set of 1,764 human transcription factor motifs for each cell.

#### Predicted enhancer–gene interactions

The activity-by-contact (ABC) model^20^ (https://github.com/broadinstitute/ABC-Enhancer-Gene-Prediction) was used for prediction of enhancer–gene interactions from scATAC-seq data. Cell-type-specific ATAC-seq signal and peak outputs from MACS2 were used as inputs. Bulk H3K27ac CUT&Tag libraries generated from similar samples (see ‘Bulk H3K27ac CUT&Tag library preparation and sequencing’ above) were used as a mark for active enhancers. Publicly available Hi-C data generated from similar samples^19^ were used to demarcate regulatory neighbourhoods, using the highest resolution available for each chromosome. Cell-type-specific gene expression profiles were generated from publicly available scRNA-seq data generated from similar samples^2^ by averaging expression across each cell type. The default threshold of 0.02 was used for calling enhancer–gene interactions.

#### VISTA enhancer intersections

VISTA enhancers were taken from the VISTA Enhancer Browser^25^ (https://enhancer.lbl.gov/) and filtered for human sequences found to be active in the forebrain. Enhancers were lifted over to Hg38 using the UCSC LiftOver tool (https://genome.ucsc.edu/cgi-bin/hgLiftOver) and overlapping regions were merged, resulting in 319 unique regions. These regions were intersected with the peak set from all primary scATAC-seq cells and 304 peaks that overlapped with VISTA forebrain enhancer regions were identified.

#### Genomic feature annotations

The ChIPSeeker R package^66^ (https://bioconductor.org/packages/release/bioc/html/ChIPseeker.html) was used to annotate all peak sets in genomic features.

#### Gene ontology

Identification of enriched biological processes in genes near to sets of cell-type-specific enhancer predictions was performed using the GREAT alogrithm^67^. For each cell type, peaks that were both predicted enhancers and cell-type-specific were identified, and enrichment of biological processes in the flanking genes of the regions relative to a background set of the full primary peak set was identified.

#### Calculating sample correlations

Correlation between samples was determined using the ‘multiBamSummary’ function from the deeptools python suite^64^ on sample bam files. Bam file comparison was limited to the genomic space of the merged primary peak set (*n* = 459,953 peaks), ignoring duplicates and unmapped reads. Heat maps were then generated using the ‘plotCorrelation’ function.

#### scRNA-seq/scATAC-seq co-embedding

To anchor mRNA expression and chromatin state profiles in the same map of cell diversity, we applied scAlign on datasets where we profiled scRNA-seq and scATAC-seq in parallel in the same sample. This was achieved by linking gene expression data to gene activity scores derived from chromatin accessibility data. The gene activity scores were logRPM values derived from gene activity scores generated by the SnapATAC pipeline. Then the gene expression and gene activity scores were processed using Seurat, and then split into batches for input into scAlign. The encoder space was computed using multi CCA of the 10 dimensions with latent dimensions at 20 using the ‘small’ architecture.

#### Pseudotime analysis

The Monocle 3 R package^68^ (https://cole-trapnell-lab.github.io/monocle3/) was used for pseudotime calculation of the co-embedded RNA and ATAC dataset. The RG cells were set as the root cells. The minimum branch length was 9 in the graph building. Monocle 3 was also used for the pseudotime calculation of the scRNA-seq PFC/V1 dataset. The Cicero package^69^ (https://cole-trapnell-lab.github.io/cicero-release/) was used for the pseudotime calculation of the scATAC-seq PFC/V1 dataset.

#### Identification of temporally dynamic peaks in the excitatory neuronal lineage

scATAC-seq cells from V1 samples used in the co-embedding analysis were divided into ten equal bins by pseudotime. Average accessibility for each peak for each bin was determined. Peaks were considered temporally dynamic if they met the following criteria: accessible in a minimum of 10% of cells in the bin with the highest accessibility; accessible in a maximum of 20% of cells in the bin with the lowest accessibility; at least a difference of 10% in proportion of cells where the peak was accessible between the lowest and highest accessibility bins; and had an increase in proportion of accessibility in cells of at least 3× between the lowest and highest accessibility bins. In total 25,415 out of 459,953 peaks met these criteria and were deemed to be temporally dynamic in the cortical excitatory neuronal lineage.

#### Comparison of accessibility, gene expression, and transcription factor motif enrichment across pseudotime

As pseudotime was calculated on the co-embedded space of ATAC and RNA cells, we can directly compare temporal changes in gene expression, gene activity scores calculated from open chromatin, and transcription factor motif enrichment. For each of the genes, we calculated gene activity scores using Cicero^69^ and calculated a 1,000-cell moving average across pseudotime from the ATAC cells. This value was normalized to represent a proportion of the maximum value. For gene expression, we calculated a 1,000-cell moving average across pseudotime from the RNA cells. This value was normalized to represent a proportion of the maximum value. For transcription factor motif enrichment, using *Z*-scores from ChromVAR, we calculated a 1,000-cell moving average of the motif enrichment across pseudotime from the ATAC cells. LOESS regression lines were fit to the moving average data. For the generation of heat maps, a similar approach was used, except values were averaged within 20 equally sized bins of pseudotime and normalized the maximum value.

#### Branchpoint analysis

URD^70^ (https://github.com/farrellja/URD/) was used to compare the branchpoints of ATAC and RNA independently. Deep-layer neurons were not considered during this analysis owing to obfuscating identities, and the batch-corrected values were used as input to the diffusion map calculations to combat batch effects. Diffusion parameters were set to 150 nearest neighbours, and sigma was autocalculated from the data. The tree was constructed using 200 cells per pseudotime bin, 6 bins per pseudotime window, and branch point *P* value threshold of 0.001.

#### Identification of homologous cell types in primary and organoid samples

To identify homologous cell types between primary and organoid scATAC-seq datasets, reads from organoid cells were counted in peaks defined in the primary dataset, providing matching peak-by-cell matrices for primary and organoid datasets. DA peaks were then identified in each dataset for each cluster as described above, and the intersection of this DA peak set was used to calculate correlations between primary and organoid clusters after averaging peak accessibility across all cells in each cluster. Homologous cell types were then determined on the basis of the highest correlation values for each cluster.

### Single-cell RNA-seq analysis

#### Seurat

For primary samples used in Figs. 2, 3, scRNA-seq data were preprocessed using a minimum of 500 genes and 5% mitochondrial cutoff and Scrublet^71^ for doublet removal. The SCTransform^72^ workflow in Seurat^73^ was run separately on each batch. Canonical component analysis (CCA) on the Pearson residuals from SCTransform was used as input into scAlign for batch correction. Dimensionality reduction and clustering were performed using PCA and Leiden, respectively, using the default parameters of the Seurat pipeline. For organoid samples used in the arealization experiment in Fig. 3, libraries from different conditions were demultiplexed using the Multi-seq pipeline (https://github.com/chris-mcginnis-ucsf/MULTI-seq). The normal SCTransform workflow was then applied, as described above. Genes that were differentially expressed between conditions were identified using the ‘FindMarkers’ function with ‘MAST’ selected as the method. For organoid samples used for validation (Extended Data Fig. 10), scRNA-seq data were integrated following the Seurat SCTransform integration workflow using default parameters.

#### Classification of area identity of organoid cells

To systematically determine whether organoid cells had a transcriptomic identity more closely aligned with human PFC or V1 cells, we implemented a previously described classifier method^42^. In brief, area gene modules defined on the basis of area-associated gene expression patterns^2,42^ were generated and module eigengene values were determined for each organoid excitatory neuron using the ‘moduleEigengenes’ function from the WGCNA R package^74^. Organoid cells were then assigned an identity of ‘PFC’ or ‘V1’ using the higher module eigengene value for each module. The significance of differences in proportions of identity labels between treatments was determined using a two-sided *χ*^2^ test (*P* < 0.05).

### Disease intersection

#### DNM enrichment

Peak sets were intersected with DNMs from 2,708 probands and 1,876 siblings using bedtools v2.24.0. DNMs were identified using an in-house pipeline. In brief, variants from whole-genome sequencing data were called using four independent callers: GATK v3.8, FreeBayes, Strelka, and Platypus. Variant calls from each caller were intersected, and filtered for read depth (>9), allele balance (>0.25), absence of reads supporting the mutation in parents, and identified by at least three of the four callers.

Sets of cell-type-specific peaks and peaks that overlapped with PLAC–seq promoter-interacting regions were tested for enrichment of DNMs in probands as compared to a background peak set which contained all primary peaks. We used Fisher’s exact test to compare the number of peaks with one or more DNMs between the cell-type-specific peak set and the background peak set. We also performed a Wilcoxon rank sum test comparing the number of DNMs per peak in the cell-type-specific set to the background peak set. We applied a Bonferroni multiple test correction to all *P* values.

#### ASD/NDD gene set enrichment

We created gene plus upstream regulatory regions using bedtools v2.24.0, where we defined the upstream regulatory region as the 100-kb region upstream of the gene transcription start sites. Gene regions were defined using Gencode V27. The total number of peaks in each gene plus upstream regulatory region was quantified per gene for each cell type and compared to the number of peaks in the merged peak set for each gene set using Fisher’s exact test. The peaks in the remaining gene plus promoter regions were used as background. Gene sets from Coe et al.^30^ (COE253), Kaplanis et al.^56^ (DDD299) and SFARI gene (https://gene.sfari.org/database/human-gene/) were used for enrichment testing. *P* values were Bonferroni corrected for multiple tests (number of peak sets).

#### Morbidity map CNV enrichment

CNVs enriched in NDD cases from Coe et al.^30^ (*n* = 70) were intersected with peak sets using bedtools 2.24.0; peaks were required to have a 50% overlap with the CNV region. The total number of peaks overlapping a CNV were compared to the number of peaks that did not overlap with a CNV for each cell type. The full primary peak set was used as background and compared by Fisher’s exact test. *P* values were Bonferroni corrected for multiple tests (number of peak sets).

#### Cell-type-specific GWAS enrichment testing

We retrieved GWAS summary statistics for schizophrenia (Ripke et al.^27^), bipolar disorder (Stahl et al.^35^), and ASD (Grove et al.^33^) from the Psychiatric Genomics Consortium data portal (https://www.med.unc.edu/pgc). We also obtained GWAS summary statistics for schizophrenia (Pardiñas et al.^32^) from http://walters.psycm.cf.ac.uk/. GWAS summary statistics for major depression (Howard et al.^34^) were obtained from the authors under the auspices of a Data Use Agreement between 23AndMe and the University of Maryland Baltimore. We applied stratified LD score regression (LDSC version 1.0.1^75,76^) to these summary statistics to evaluate the enrichment of trait heritability in each of ten predicted enhancer sets. These associations were adjusted for the union of the peak sets as well as for 52 annotations from version 1.2 of the LDSC baseline model (including genic regions, enhancer regions and conserved regions^76^). Associations that met a cutoff of FDR <0.05 were considered significant.

#### TAD enrichment

Odds ratios were calculated as the likelihood of a TAD containing an ATAC peak if it also contained a gene from the set denoted by the subplot title, with significance identified using a Fisher’s exact test. The magenta dotted line indicates a significance threshold of *P* < 0.05. Gene sets were obtained from http://resource.psychencode.org/^24,31^. TAD sets were from human brain, germinal zone (GZ) and cortical plate (CP)^19^.

### Immunohistochemistry

Samples used for immunostaining were fixed in 4% PFA for 45 min, washed out with PBS, and incubated overnight in a 30% sucrose solution at 4 °C. Samples were then embedded in a 1:1 solution of OCT and 30% sucrose and frozen at −80 °C until ready for sectioning. Cryosections were prepared at a thickness of 16 μm. Heat-induced antigen retrieval was performed in 10 mM sodium citrate (pH 6.0) for 15 min. Permeabilization was performed in PBS (pH 7.4) supplemented with 2% Triton X-100. Primary and secondary antibodies were diluted and incubated in PBS (pH 7.4) supplemented with 10% donkey serum, 2% Triton X-100, and 0.2% gelatin. Primary antibodies used in this study included: mouse anti-AUTS2 (1:200, Abcam ab243036), rabbit anti-NR2F1 (1:100, Novus Biologicals NBP1-31259), mouse anti-SATB2 (1:250, Santa Cruz Biotechnology SC-81376), rat anti-CTIP2 (1:500, abcam AB18465), rabbit anti-FOXG1 (1:500, Abcam ab196868), and rabbit anti-PAX6 (1:200, Biolegend 901301). Secondary antibodies used were AlexaFluor secondary antibodies. Images were collected using Leica SP5 confocal system and processed using ImageJ/Fiji.

### Reporting summary

Further information on research design is available in the Nature Research Reporting Summary linked to this paper.

## Online content

Any methods, additional references, Nature Research reporting summaries, source data, extended data, supplementary information, acknowledgements, peer review information; details of author contributions and competing interests; and statements of data and code availability are available at 10.1038/s41586-021-03209-8.

## Supplementary information

Supplementary Information This file contains Supplementary Table Legends for tables 1-25.

Reporting Summary

Supplementary Table This file contains Supplementary Table 1.

Supplementary Table This file contains Supplementary Table 2-13.

Supplementary Table This file contains Supplementary Table 14.

Supplementary Table This file contains Supplementary Table 15.

Supplementary Table This file contains Supplementary Table 16 and 17.

Supplementary Table This file contains Supplementary Table 18.

Supplementary Table This file contains Supplementary Table 19.

Supplementary Table This file contains Supplementary Table 20.

Supplementary Table This file contains Supplementary Table 21.

Supplementary Table This file contains Supplementary Table 22.

Supplementary Table This file contains Supplementary Table 23.

Supplementary Table This file contains Supplementary Table 24.

Supplementary Table This file contains Supplementary Table 25.

## Data Availability

scATAC-seq and scRNA-seq data derived from primary human samples are available on the NeMO archive (https://assets.nemoarchive.org/dat-gnot1gb) and the psychENCODE Knowledge Portal (https://www.synapse.org/#!Synapse:syn21392931). scATAC-seq and scRNA-seq data derived from cortical organoids are also available on the psychENCODE Knowledge Portal (https://www.synapse.org/#!Synapse:syn21392931) and GEO (GSE163018). Peak level scATAC-seq primary and organoid ATAC-seq data are available through the UCSC Cell Browser (https://cortex-atac.cells.ucsc.edu/) and UCSC Genome Browser (https://urldefense.proofpoint.com/v2/url?u=https-3A__genome.ucsc.edu_s_Max_cortex-2Datac&d=DwIBaQ&c=iORugZls2LlYyCAZRB3XLg&r=wIGwA13tJ0H_yBH_8fGR_aHDv_Lb9BdBvaGRmKuMfC8&m=C-AKivMuKdU2JxBfFMIkS53e2NDAh9SJrG2tdmW5_MU&s=Sg2BoS6TTUoAMLyXiaM6hGHhNtG9LqaUBpXoPQxWBuQ&e=).

## Code availability

Custom codes used in this study are available at the following GitHub repository: https://github.com/NOW-Lab/scATACcortex.
