# Supplementary material for: Single-cell epigenomics reveals mechanisms of human cortical development
Source: Nature. 2021 Oct 6;598(7879):205–13. doi: 10.1038/s41586-021-03209-8 (PMC8494642; doi:10.1038/s41586-021-03209-8)
Supplement: Supplementary file 1 — This file contains Supplementary Table Legends for tables 1-25. [file 41586_2021_3209_MOESM1_ESM.pdf]

nature portfolio

https://doi.org/10.1038/s41586-021-03209-8

Supplementary information

Single-cell epigenomics reveals mechanisms of human cortical development

In the format provided by the authors and unedited

Supplementary Table Legends

Supplementary Table 1. Sample metadata and key summary statistics for all scATAC-seq samples including sequencing depth, cell counts, fraction of fragments in peaks, and fraction of fragments in promoters.

Supplementary Table 2. All primary scATAC-seq peaks.

Supplementary Table 3. MACS2 scATAC-seq peaks by broad cell type

Supplementary Table 4. Cell type specific scATAC-seq peaks by broad cell type (Fisher's Exact, two-sided, FDR < 0.05)

Supplementary Table 5. ABC Predicted enhancer-gene interactions by broad cell type (Using activity-by-contact method)

Supplementary Table 6. Cluster specific scATAC-seq peaks – primary (Fisher's Exact, two-sided, FDR < 0.05)

Supplementary Table 7. CellWalker cell type specific peaks (Fisher's Exact, two-sided, FDR < 0.05)

Supplementary Table 8. Area specific peaks (Fisher's Exact, two-sided, FDR < 0.05)

Supplementary Table 9. Temporally dynamic peaks in excitatory neurogenesis

Supplementary Table 10. PFC>V1 DA peaks (Fisher's Exact, two-sided, FDR < 0.05)

Supplementary Table 11. V1>PFC DA peaks (Fisher's Exact, two-sided, FDR < 0.05)

Supplementary Table 12. oRG>tRG DA peaks (Fisher's Exact, two-sided, FDR < 0.05)

Supplementary Table 13. tRG>oRG DA peaks (Fisher's Exact, two-sided, FDR < 0.05)

Supplementary Table 14. 25-state chromatin model intersection – primary scATAC-seq peaks; using 25-state imputed model from the Roadmap Epigenomics Project (E081)

Supplementary Table 15. Public dataset intersections – primary scATAC-seq peaks

Supplementary Table 16. All organoid scATAC-seq peaks

Supplementary Table 17. Cluster specific scATAC-seq peaks – organoid (Fisher's Exact, two-sided, FDR < 0.05)

Supplementary Table 18. Primary/Organoid scATAC-seq peak intersection

Supplementary Table 19. Public dataset intersections – organoid scATAC-seq peaks

Supplementary Table 20. HOMER motif enrichments in cell type specific scATAC-seq peak sets (Hypergeometric test, one sided)

Supplementary Table 21. HOMER motif enrichments in PFC>V1 and V1>PFC scATAC-seq peak sets (Hypergeometric test, one sided)

Supplementary Table 22. HOMER motif enrichments in oRG>tRG and tRG>oRG scATAC-seq peak sets (Hypergeometric test, one sided)

Supplementary Table 23. HOMER motif enrichments in organoid scATAC-seq peaks not present in primary scATAC-seq dataset (Hypergeometric test, one sided).

Supplementary Table 24. PFC/V1 IPC DE genes (Adjusted P-value < 0.05, using Seurat MAST method, two-sided)

Supplementary Table 25. PFC>V1 and V1>PFC IPC DA peaks (FDR<0.05, Fisher's Exact, two-sided)
