# Supplementary material for: Single-cell epigenomics reveals mechanisms of human cortical development
Source: Nature. 2021 Oct 6;598(7879):205–13. doi: 10.1038/s41586-021-03209-8 (PMC8494642; doi:10.1038/s41586-021-03209-8)
Supplement: Supplementary file 2 — Reporting Summary [file 41586_2021_3209_MOESM2_ESM.pdf]

nature research

Corresponding author(s): Tomasz J. Nowakowski

Last updated by author(s): Dec 10, 2020

Reporting Summary

Nature Research wishes to improve the reproducibility of the work that we publish. This form provides structure for consistency and transparency in reporting. For further information on Nature Research policies, see Authors & Referees and the Editorial Policy Checklist.

Statistics

For all statistical analyses, confirm that the following items are present in the figure legend, table legend, main text, or Methods section.

n/a	Confirmed	
☐	☒	The exact sample size (*n*) for each experimental group/condition, given as a discrete number and unit of measurement
☐	☒	A statement on whether measurements were taken from distinct samples or whether the same sample was measured repeatedly
☐	☒	The statistical test(s) used AND whether they are one- or two-sided<br>*Only common tests should be described solely by name; describe more complex techniques in the Methods section.*
☐	☒	A description of all covariates tested
☐	☒	A description of any assumptions or corrections, such as tests of normality and adjustment for multiple comparisons
☐	☒	A full description of the statistical parameters including central tendency (e.g. means) or other basic estimates (e.g. regression coefficient) AND variation (e.g. standard deviation) or associated estimates of uncertainty (e.g. confidence intervals)
☐	☒	For null hypothesis testing, the test statistic (e.g. *F*, *t*, *r*) with confidence intervals, effect sizes, degrees of freedom and *P* value noted<br>*Give P values as exact values whenever suitable.*
☒	☐	For Bayesian analysis, information on the choice of priors and Markov chain Monte Carlo settings
☒	☐	For hierarchical and complex designs, identification of the appropriate level for tests and full reporting of outcomes
☐	☒	Estimates of effect sizes (e.g. Cohen's *d*, Pearson's *r*), indicating how they were calculated

Our web collection on statistics for biologists contains articles on many of the points above.

Software and code

Policy information about availability of computer code

Data collection	No software was used for data collection.
Data analysis	Open source software including Cell Ranger v2, Cell Ranger ATAC v1, SnapTools v1.4, deeptools v3.1.3, HOMER v4.11, MACS2 v2.1.1.20160309, Activity-by-Contact v0.2, GATK v3.8, FreeBayes v1.1.0, Strelka v2.9.2, Platypus v0.8.1, bedtools v2.24.0, LDSC v1.0.1, R v3.6.3, and the R packages: SnapATAC v1.0.0, scAlign (git commit ID: 40a1a5caddc262afe5f2ef2119e0e1773162aecf), Seurat v3.1.5, monocle3 v0.2.2.0, Cicero v1.4.4, URD v1.1.0, ChIPseeker v1.22.1, rGREAT 1.18.0, chromVAR v1.8.0, liftOver v1.10.0, CellWalkR v0.1.7, and WGCNA 1.69 were used in this study for data analysis. Custom code used in this study are available in the following GitHub repository: https://github.com/NOW-Lab/scATACcortex

For manuscripts utilizing custom algorithms or software that are central to the research but not yet described in published literature, software must be made available to editors/reviewers. We strongly encourage code deposition in a community repository (e.g. GitHub). See the Nature Research guidelines for submitting code & software for further information.

Data

Policy information about availability of data

All manuscripts must include a data availability statement. This statement should provide the following information, where applicable:

- Accession codes, unique identifiers, or web links for publicly available datasets
- A list of figures that have associated raw data
- A description of any restrictions on data availability

scATAC-seq and scRNA-seq data derived from primary human samples are available on the NeMO archive (https://assets.nemoarchive.org/dat-gnot1gb) and the psychENCODE Knowledge Portal (https://www.synapse.org/#!Synapse:syn21392931). scATAC-seq and scRNA-seq data from derived from cortical organoids are also available on the psychENCODE Knowledge Portal (https://www.synapse.org/#!Synapse:syn21392931) and GEO (GSE163018). Peak level scATACseq primary and organoid ATAC-seq data is available through the UCSC Cell Browser (https://cortex-atac.cells.ucsc.edu/) and UCSC Genome Browser (https://urldefense.proofpoint.com/v2/url?

u=https-3A__genome.ucsc.edu_s_Max_cortex-2Datac&d=DwIBaQ&c=iORugZls2LlYyCAZRB3XLg&r=wIGwA13tJ0H_yBH_8fGR_aHDv_Lb9BdBvaGRmKuMfC8&m=C-AKivMuKdU2JxBfFMIkS53e2NDAh9SJrG2tdmW5_MU&s=Sg2BoS6TTUoAMLyXiaM6hGHhNtG9LqaUBpXoPQxWBuQ&e=).

Field-specific reporting

Please select the one below that is the best fit for your research. If you are not sure, read the appropriate sections before making your selection.

☒ Life sciences ☐ Behavioural & social sciences ☐ Ecological, evolutionary & environmental sciences

For a reference copy of the document with all sections, see nature.com/documents/nr-reporting-summary-flat.pdf

Life sciences study design

All studies must disclose on these points even when the disclosure is negative.

Sample size	Sample size for primary human scATAC-seq data was chosen based on the number of distinct individuals for which samples were available (n=6). For organoid scATAC-seq data generation n=3 distinct lines were used. No statistical methods were used to determine sample size.
Data exclusions	Low quality cells were excluded from primary human and organoid scATAC-seq datasets as described in Methods.
Replication	For primary samples, we performed our experiments on specimens from 6 individuals. For cerebral organoids, we performed our experiments on 3 different lines. All replication attempts were successful.
Randomization	Randomization was not used in this study.
Blinding	Investigators were not blinded in this study as quantitative measures were used to measure our results.

Reporting for specific materials, systems and methods

We require information from authors about some types of materials, experimental systems and methods used in many studies. Here, indicate whether each material, system or method listed is relevant to your study. If you are not sure if a list item applies to your research, read the appropriate section before selecting a response.

Materials & experimental systems

n/a	Involved in the study	
☐	☒	Antibodies
☐	☒	Eukaryotic cell lines
☒	☐	Palaeontology
☒	☐	Animals and other organisms
☐	☒	Human research participants
☒	☐	Clinical data

Methods

n/a	Involved in the study	
☒	☐	ChIP-seq
☒	☐	Flow cytometry
☒	☐	MRI-based neuroimaging

Antibodies

Antibodies used	mouse anti-AUTS2 (1:200, Abcam ab243036), rabbit anti-NR2F1 (1:100, Novus Biologicals NBP1-31259), mouse anti-SATB2 (1:250, Santa Cruz Biotechnology SC-81376), rat anti-CTIP2 (1:500, abcam AB18465), rabbit anti-FOXG1 (1:500, Abcam ab196868), and rabbit anti-PAX6 (1:200, Biolegend 901301). Secondary antibodies used were AlexaFluor secondary antibodies.
Validation	Only previously published antibodies or antibodies with company based validation were used. Website of each company can be consulted.

Eukaryotic cell lines

Policy information about cell lines

Cell line source(s)	PSC lines (1323-4, H28126, and H1) were obtained from our collaborators in Arnold Kriegstein's lab at UCSF. Original sources: 1323-4 - Gladstone Institutes, H28126 - University of Chicago, H1 - WiCell
Authentication	Lines were obtained using MTA approval; at time of generation lines were karyotyped for normal identity.
Mycoplasma contamination	Lines are negative for mycoplasma.
Commonly misidentified lines (See ICLAC register)	None.

Human research participants

Policy information about studies involving human research participants

Population characteristics	Human tissue samples were collected without any identifying information including sex and race.
Recruitment	No human participants were involved in this study. Human tissue samples were obtained from elective terminations with patient's prior consent.
Ethics oversight	All primary tissue was obtained and processed as approved by the UCSF Human Gamete, Embryo, and Stem Cell Research Committee (GESCR).

Note that full information on the approval of the study protocol must also be provided in the manuscript.
